# Splice-altering *TP53* missense mutations as drivers of dysfunction and targets for RNA-based therapy

**DOI:** 10.1186/s13287-026-05176-1

**Published:** 2026-07-13

**Authors:** Sun-Ku Chung, Han-Byul Jung, Su-Jin Baek, Sang-Min Park, Jinchul Kim, Seo-Young Lee, Byungkuk Min, Yong-Kook Kang

**Affiliations:** 1 Division of KM Science Research, Korea Institute of Oriental Medicine, Daejeon, 34054 Republic of Korea; 2 Korean Convergence Medical Science, KIOM Campus, University of Science and Technology, Daejeon, 34054 Republic of Korea; 3 Future Medicine Division, Korea Institute of Oriental Medicine, Daejeon, 34054 Republic of Korea; 4 College of Pharmacy, Chungnam National University, Daejeon, 34134 Republic of Korea; 5 Ageing Research Center, Korea Research Institute of Bioscience and Biotechnology, Daejeon, 34141 Republic of Korea; 6 Development and Differentiation Research Center, Aging Convergence Research Center (ACRC), Korea Research Institute of Bioscience Biotechnology (KRIBB), Daejeon, 34141 Republic of Korea

**Keywords:** p53 pathway, Cancer genomics, RNA therapeutics, Splicing modulation, *TP53* mutation

## Abstract

*TP53* missense mutations introduce single amino acid substitutions in the p53 protein, which can lead to diverse functional consequences. However, their post-transcriptional impact, particularly on RNA splicing, remains underexplored. Herein, we analyzed cancer genome databases and identified 34 *TP53* missense and synonymous mutations capable of generating *de novo-*splice sites. Using minigene assays, we confirmed the splicing-altering potential of several mutations, including c.178 C > A, c.182 A > G, c.318 C > G, c.356 C > G, c.362 C > A, c.551 A > G, and c.922 C > G. To assess the physiological relevance of these splicing changes, we developed mutant *TP53* knock-in cell models using bacterial artificial chromosome DNA-mediated homologous recombination. These missense mutations can function as frameshift or hypomorphic mutations due to aberrant splicing, significantly compromising *TP53* mRNA integrity. Meanwhile, severe impairment of constitutive splicing suggests that it correlates with an increased likelihood of nonsense-mediated mRNA decay for mutation-driven alternative transcripts carrying a frameshifted premature stop codon. By designing antisense morpholino oligomers targeting *de-novo* splice site, we were able to restore csV1 expression and enhance p53 function. This reclassification of select p53 mutations from simple missense to splicing-disruptive mutations reveals their underlying loss-of-function mechanisms and highlights their therapeutic reversibility. Our findings present a novel framework for RNA-based therapeutic strategies aimed at correcting splicing defects in *TP53*-mutant cancers.

**Supplementary Information:**

The online version contains supplementary material available at https://doi.org/10.1186/s13287-026-05176-1.

## Background

The deletion or insertion of one or more nucleotides within the exonic region of *TP53* can disrupt the reading frame, resulting in a frameshift mutation [1–5]. In a frameshift mutation, the addition or deletion of specific sequences is readily detectable. Therefore, it is reasonable to expect that an erroneous reading frame will be generated. However, the potential for substitution mutations to cause similar disruptions has inadvertently been overlooked, with studies typically focusing on missense mutations that result in prominent amino acid changes. Empirical assumptions suggest that mutations in the exonic region of the *TP53* gene are commonly categorized as missense mutations, which lead to amino acid substitutions. However, focusing exclusively on functional studies at the protein level limits the ability to explore the broader effects of mutations in the *TP53* exonic regions beyond the translational stage, limiting the scope of research on mutant p53. A typical missense mutation, *TP53* c.359 A > G, results in a lysine-to-arginine substitution. This substitution affects a lysine residue essential for p53 acetylation, impairing protein acetylation and reducing tumor suppression [6, 7]. However, our analysis of c.359 A > G knock-in cells revealed negligible levels of both the mutant p53 protein and its transcript, prompting an investigation into pre-translational regulatory mechanisms [8, 9]. Overall, we demonstrated that this mutation, rather than primarily affecting protein function via amino acid substitution, creates a novel splicing donor site that severely disrupts constitutive *TP53* mRNA processing.

Building on our previous studies [8], we expanded the scope of our research by investigating a broader array of mutations, leveraging cancer genome databases to identify missense mutations that may directly create novel splice sites, a previously underexplored feature. To confirm that candidate mutations derived from splicing prediction tools contributed to the creation of novel splice sites, we used minimal gene fragments containing individual mutations, including *TP53* c.178 C > A, *TP53* c.182 A > G, *TP53* c.318 C > G, *TP53* c.356 C > G, *TP53* c.362 C > A, *TP53* c.551 A > G, and *TP53* c.922 C > G. Furthermore, through genetic recombination [10–12], we generated knock-in cells harboring *TP53* variants previously shown by minigene assays to affect splicing, including c.182 A > G, c.318 C > G, c.356 C > G, and c.362 C > A. In addition to these genetically engineered cells, we identified and characterized cancer cell lines harboring *TP53* mutations that directly affect splicing. Based on these findings, we investigated the presence of novel variants generated by each mutation-generated splice site and examined their impact on constitutive splice sites. We also assessed the qualitative and quantitative impacts of these mutations on the expression of *TP53* variants and their subsequent effects on p53 functionality in cells. Furthermore, by studying various splicing modulators, such as antisense morpholino oligomers, which can directly target and interfere with splice sites [9, 13, 14], we elucidated the role of mutations in the creation of *de novo*-splice sites and their potential to cause frameshift or hypomorphic mutations, leading to functional loss or deficiency [15].

Even when the RNA-level consequences of *TP53* substitution mutations are precisely identified and normal splicing is restored through the use of splicing modulators, enabling the synthesis of p53 protein, the therapeutic potential of these modulators as RNA-targeted treatments can only be fully evaluated once the functional consequences of the mutation-mediated amino acid substitutions are thoroughly understood. In other words, restoring adequate protein levels through splicing modulation may enable certain loss-of-function or hypomorphic *TP53* variants to regain partial p53 activity, potentially restoring aspects of wildtype p53 function. Accordingly, we classified the identified mutations into distinct categories, such as hypomorphic or gain-of-function mutations, to better elucidate their functional implications.

## Materials and methods

### Construction of minigenes

To construct minigenes, sequences containing each *TP53* mutation were synthesized by PCR fragments carrying each *TP53* mutation were generated using mutagenic primer pairs and 10 ng of the BAC clone RP11-199F11 from the BACPAC Resources Center as the template. To construct a minigene for the c.178 C > A mutation, PCR amplification was performed with the primer pairs 5cF/178R or 178 F/5cR, respectively (see Supplementary Table 6). PCR was performed for 36 cycles at 95 °C (1 min), 58° (1 min), and 72 °C (1 min) using LongAmp^®^ *Taq* DNA Polymerase (New England Biolabs). The PCR-amplified fragments were purified using the QIAquick PCR purification kit (New England Biolabs). For application to the In-Fusion Cloning system (Takara), the ends of the purified fragments are composed of homologous sequences so that each fragment can be fused, and the ends of the two fused fragments are also composed of homologous sequences so that they can be fused with the pcDNA3.1+/C-DYK vector linearized by EcoRV restriction endonuclease. According to the protocol of the In-Fusion Cloning system, the mutant fragments were inserted into the EcoRV restriction endonuclease site of the pcDNA3.1+/C-DYK vector by fusing each fragment with the linearized vector. The constructs were finally confirmed by DNA sequence analysis. Similarly, the following primer pairs were used to generate other mutations: c.182 A > G mutation: 5cF/182R, 182 F/5cR; c.318 C > G mutation: 5cF/318R, 318 F/5cR; c.356 C > G mutation: 5cF/356R, 356 F/5cR; c.362 C > A mutation: 5cF/362R, 362 F/5cR; c.551 A > G mutation: 551F1/551R1, 551F2/551R2, 551F3/551R3; and c.922 C > G mutation: 992F1/992R1, 992F2/992R2, 992F3/992R3 (see Supplementary Table 6).

### Fragment analysis from minigenes

Using Gene Pulser Xcell™ Electroporation System (Bio-Rad), 1 µg of minigene constructs were transfected into 2 × 10^5^ H1299 cells (ATCC), respectively, under the following conditions: 250 V and 200 µF. The cells were maintained in Roswell Park Memorial Institute medium 1640 (RPMI 1640) supplemented with 10% Fetal Bovine Serum, 1% penicillin/streptomycin (Thermo Scientific). After electroporation, 48 h later, the cellular RNA preparation, cDNA synthesis and PCR reaction were performed like below “Quantitative real-time PCR and semiquantitative RT-PCR method”. PCR amplicons were amplified by the primer pairs described in Supplementary Table 7. DNA fragment analysis was performed using the dsDNA 915 Reagent Kit (DNF-915-22, Agilent) on a Fragment Analyzer system (Advanced Analytical Technologies, Device Serial #4007). DNA samples were prepared according to the manufacturer’s protocol, and electrophoresis was carried out using a 12-capillary array filled with a pre-formulated gel matrix. Fluorescent intercalating dye included in the reagent kit was used for DNA detection. The dye binds specifically to double-stranded DNA (dsDNA), enhancing fluorescence upon binding. The fluorescence signal was recorded as the DNA fragments passed through the detection window, and fragment sizes were determined using the ProSize 2.0 software (Agilent Technologies). Internal size markers (35 bp and 5000 bp) and a 100 bp DNA ladder were used as references to ensure accurate fragment size estimation and quantification. Data were analyzed to determine the fragmentation patterns, and samples were identified using DNA sequencing analysis. Electrophoresis was performed under the following conditions: a pre-run step at 7.0 kV for 30 s, followed by marker and sample injection at 4.5 kV for 5 s. The separation was carried out at 7.0 kV for 24 min.

### Construction of bacterial artificial chromosome (BAC)-based targeting vectors

Previously, the *TP53* genome, including partial intron 1 to partial intron 7, was inserted between the *Nhe*I and EcoRV restriction endonuclease sites of the pcDNA3.1 + vector. Based on the vector, the selection cassette (FRT-flanked CAG promoter-neo resistance gene) was inserted into the *Hpa*I restriction endonuclease site in the intron 1 of the *TP53* genome. The c.182 A > G, c.318 C > G, c.356 C > G, or c.362 C > A mutations for BAC construction are flanked by *Psh*AI restriction enzyme sites. Therefore, instead of the fragment removed from the *TP53* genome by the *Psh*AI restriction endonuclease, a sequence fragment of the same length including the mutation of the minigene construct was inserted using the Quick Ligation™ kit. Plasmids containing the selection cassette were constructed by replacing the c.182 A > G, c.318 C > G, c.356 C > G, or c.362 C > A mutation sites located between the two *Psh*AI restriction sites within the *TP53* genomic sequence, respectively. To insert the sequence containing the selection cassette and each *TP53* mutation into BAC DNA, the DNA fragments were linearized with *Nhe*I and *Pml*I restriction endonucleases to have 0.3 kb and 1.5 kb homology arms on either side of the selection cassette, and then electroporated into the SW102 strain containing RP11-199F11.

### Generation of *TP53* mutant cells

Previously characterized Human induced Pluripotent Stem Cells (hiPSCs) were cultured in mTeSR™ Plus medium (STEMCELL technologies). For passaging hiPSCs, the confluent cells were washed with D-PBS (Invitrogen) and trypsinized with Accutase™ (STEMCELL technologies). 100 µg of BAC DNA linearized by *Not*I restriction endonuclease was electroporated into 20 × 10^6^ hiPSCs. Electroporation was conducted with a Gene Pulser Xcell™ Electroporation System (Bio-Rad) under the following conditions: 250 V and 200 µF. After electroporation, 2 d later, the transfected hiPSCs were selected with G418 (50 µg/ml) for 12 d. Surviving colonies were individually picked and expanded. To screen the heterozygous knock-in hiPSC clones, genomic DNA was extracted from hiPSC clones as previously described [8]. The G1 and G3 primers for the genotype described in Supplementary Table 7 were aligned outside the selection cassette or heterozygous polymorphic marker, *rs12944939*. PCR amplicons were produced by these primer pairs, digested by *Aci*I restriction endonuclease (New England Biolabs), and then run on a 2% agarose gel. The selection cassette (FRT-flanked CAG promoter-neo resistance gene) was removed from the heterozygous mutant cells by Flippase (FLP) recombinase. To generate homozygous mutant knock-in hiPSCs, the same procedure used to generate heterozygous mutant knock-in hiPSCs from wildtype hiPSCs was repeatedly applied to heterozygous mutant knock-in hiPSCs. To analyze the homozygous mutant knock-in hiPSC clones, genomic DNA was amplified by PCR with the three primers mixture (G1 + G2+G3) described in Supplementary Fig. 2A, B and Supplementary Table 7.

### Cell culture

SNU-368 hepatocellular carcinoma cell line was obtained from Korea Cell Line Bank (Seoul National University, Seoul, South Korea). SKN-3 oral cavity squamous carcinoma cell line was obtained from JCRB Cell Bank (National Institutes of Biomedical Innovation, Health and Nutrition, Osaka, Japan). These cell lines were maintained in RPMI 1640 supplemented with 10% Fetal Bovine Serum, 1 × non-essential amino acids (NEAA, Thermo Fisher Scientific), 0.1 mM β-mercaptoethanol (β-ME, Thermo Fisher Scientific), penicillin/streptomycin (Thermo Fisher Scientific).

### Construction of mutant U1 (U1^mut^) plasmid

The pRGEN-U6 vector (Bioneer) deleted the region from the *Nde*I site of the internal U6 promoter to the *Nru*I restriction site of the external tracrRNA and replaced it with 393 bp of the U1 promoter, 164 bp of mutant U1, and 39 bp of the 3´-flanking region. a total of 596 bp from the U1 promoter to the 3´-flanking region was generated by gene synthesis along with U1 mutagenesis (see Supplementary Fig. 3A, B). Using Gene Pulser Xcell™ Electroporation System (Bio-Rad), 2 µg of U1^mut^ constructs were transfected into 5 × 10^5^ *TP53*-mutant hiPSC cells, respectively, under the following conditions: 250 V and 200 µF. After electroporation, 2d later, they were provided for RNA or protein extraction, which is described in detail in “Quantitative real-time PCR” or “Western blot” methods below.

### Prediction of splicing affecting mutations

The mutation information of TP53 were integrated from The Cancer Genome Atlas (TCGA) (https://www.cancer.gov/tcga), Catalogue of Somatic Mutations in Cancer (COSMIC) (https://cancer.sanger.ac.uk/cosmic), and Universal Mutation Database (UMD) (https://p53.fr/tp53-database). Only missense and synonymous mutations categorized as “Missense_Mutation” in TCGA, “Substitution - Missense” and “Substitution - coding silent” in COSMC, and “Missense_Mutation” and “Synonymous_Mutation” in UMD were selected for the following analysis. The effect of selected mutations was evaluated by NetGene2 (https://services.healthtech.dtu.dk/services/NetGene2-2.42/) and MutPred Splice (http://mutdb.org/mutpredsplice). The prediction results from NetGene2 were categorized according to their impact: (1) ‘creation of new site’ if the mutation created a new site for splicing, (2) ‘loss of existing site’ if the mutation blocked a pre-existing site for splicing, and (3) ‘changes in probability of existing sites’ if the mutation increased or decreased the probability of splicing at a pre-existing site. In each mutation, both changes in the likelihood of the splicing site acting as a donor or acceptor were predicted. The selection threshold for the mutations was 0.3 for NetGene2’s confidence level. The MutPred’s score of 0.2 was set as an additional threshold for the selected mutations.

### Sashimi plots

For detection and quantification of alternative splicing events, reads in a BAM file of each *TP53*-mutant hiPSC were mapped on a pre-built human reference transcripts (hg19) using rMATS-turbo v4.3.0 [16] with enabled --novelSS and --individual-counts, while setting --cstat to 0. The rMATS results were visualized by ‘rmats2sashimiplot v3.0.0 (https://github.com/Xinglab/rmats2sashimiplot)’, and finally Sashimi plots representing the mutation-mediated alternative splicing events at target regions were selected.

### RNA extraction and RNA-sequencing

Total RNA was extracted using TRIzol^®^ RNA Isolation Reagents (Life technologies, Carlsbad, CA, USA). The quantity and quality of the total RNA were assessed using the Agilent 2100 bioanalyzer RNA kit (Agilent technologies, CA, USA). Total RNA was processed to prepare an mRNA-sequencing library using the Illumina TruSeq Stranded mRNA sample preparation kit (Illumina, San Diego, CA, USA) according to the manufacturer’s protocol. The quality and fragment size distribution of the libraries were evaluated using the Agilent 2100 Bioanalyzer DNA kit (Agilent technologies, CA, USA). Libraries were quantified by qPCR using CFX96 Real-Time System (Bio-Rad, Hercules, CA, USA) and sequenced on the DNBSEQ-G400 sequencers (MGI) with paired-end 150 bp reads.

### RNA-sequencing data analysis

The reads were aligned to the human reference genome (hg19) using the Tophat2 aligner [17]. Read count was calculated as gene expression abundance using cufflinks [18] with Ensembl gene models. The expression difference of the AMO-treated p53 variants (AMO group) and the AMO-untreated group (Control group) in terms of change rate (fold change; FC) and statistical significance (*p* < 0.05) were analyzed using edgeR [19]. For each p53 mutations, genes sowing at least a four-fold change in expression before and after doxorubicin treatment —based on log2-transformed count values— were identified as differentially expressed genes (DEGs). Gene set enrichment analysis (GSEA) was performed using the fgsea (v1.12.0) package in Bioconductor on a pre-ranked gene list generated from differentially expressed gene (DEG) analysis (ranked by log2 fold change). Hallmark gene sets were obtained from the Molecular Signatures Database (MSigDB) and used as the reference gene sets [20].

### Correlation analysis of expression of p53 variants and p53 target genes

To evaluate the relationship between *TP53* and its target genes, we performed correlation analysis between the expression level of *TP53* and the expression levels of individual *TP53* target genes across all samples. Gene expression values were obtained from Cufflinks-generated counts and log2-transformed prior to analysis. A curated list of canonical *TP53* target genes was obtained from the TRRUST database [21], and a total of 106 unique genes were included after excluding those annotated as “Unknown” targets. Pearson correlation coefficients and corresponding p-values were calculated using the scipy.stats.pearsonr function. Statistical significance was determined at *p* < 0.05, only genes showing significant correlations were included in downstream analysis. Target genes that were significantly co-expressed with the p53 variants were visualized using dot plots and line plots generated with the ggpubr and ggplot2 packages in R. Functional enrichment analysis of the identified target genes was performed using Enrichr [22], based on Hallmark gene sets. The pathway-target gene network was constructed using cytoscape (v.3.10.0) [23], providing a comprehensive pathway-target gene associations.

### Antisense morpholino oligonucleotide (AMO) and small interfering RNA (siRNA)

The AMOs listed in Supplementary Table 7 were purchased from Gene Tools. The 20 µM AMO transfection was performed with Endo-porter (Gene tools) according to the manufacturer’s instructions.

The siRNA against UPF1 (Silencer^®^ Select #s11928) and that used for the negative control (Silencer^®^ Select negative control 1) were purchased from Thermo Scientific. A total of 5 × 10^5^ *TP53*-mutant hiPSCs were transfected with 10 µM siRNA using a Gene Pulser Xcell™ Electroporation System (Bio-Rad) under the following conditions: 250 V and 200 µF.

### Quantitative real-time PCR

Cellular RNA was isolated using the RNeasy Mini Kit (QIAGEN) according to the manufacturer’s instructions. cDNA was synthesized from 2 µg of total RNA using the Superscript III First Strand Synthesis System for RT-PCR (Invitrogen). For cDNA synthesis, 2 µg of total RNA was reverse transcribed using the SuperScript™ III First-Strand Synthesis System (#18080-051, Invitrogen) with random hexamer according to the manufacturer’s protocol. The reaction conditions were as follows: 25 °C for 10 min, 50 °C for 50 min, followed by enzyme inactivation at 85 °C for 5 min. cDNA was diluted to a constant concentration. Quantitative real-time PCR (qPCR) was performed using the iTaq™ Universal SYBR^®^ Green Supermix (#1725121, Bio-rad) on CFX96 Real-time system (Bio-rad) under following conditions: 40 cycles of DNA denaturation at 95 °C for 15 s, and annealing/polymerization at 60 °C for 1 min. Relative gene expression levels were analyzed using the ∆∆Ct method, with *GAPDH* as the endogenous control. All reactions were performed in technical triplicates to ensure reproducibility. Primer sequences were summarized in Supplementary Table 7.

### Western blot

For western blotting, cell lysate was prepared using a Cell Lysis Buffer II (#FNN0021, Invitrogen, ) with cOmplete™ ULTRA Tablets, Mini, EASYpack Protease Inhibitor Cocktail (Roche). Protein concentrations were quantified using the Pierce™ Rapid Gold BCA Protein Assay Kit (#A53225, Thermo Fisher Scientific). Proteins were boiled in SDS loading buffer (50 mM Tris-HCl (pH 6.8), 2% SDS, 6% glycerol, 0.004% bromophenol blue, 5% β-mercaptoethanol). After heat denaturation, 10–20 µg of protein extracts were resolved by SDS gel electrophoresis on 4–15% Mini-PROTEAN^®^ TGX™ Precast Protein Gels (#4561084, Bio-rad), and transferred to PVDF membranes (#1620177, Bio-rad) or nitrocellulose membrane (#10600003, GE Healthcare Life science). Membranes blocked using 5% skim milk (#232100, BD Difco) or 5% BSA (#A7030, Roche) in TBST (EBA-1101T, Elpis) and probed with p53 (DO-7) (#48818S, Cell signaling), p21 (#2947, Cell signaling), Puma (sc374223, Santacruz), Bax (#2772S, Cell signaling) or β-actin (#sc47778, Santacruz) for 16 h at 4°C. Primary antibodies were diluted in TBST as follows: Puma at 1:200, and all other primary antibodies at 1:1000. HRP-conjugated Goat anti-mouse IgG F(ab’)2, polyclonal antibody (#ADI-SAB-100 J, Enzo) or goat-anti-rabbit (#ADI-SAB-300 J, Enzo) were diluted in 5% BSA at 1:5000 and added for one hour at RT. SuperSignal™ West Dura Extended Duration Substrate (#17-1164734, Thermo Fisher Scientific) was used for detection on GE ImageQuant LAS-4000 (GE Healthcare Bio-sciences Corp.).

### Apoptosis and proliferation assay

To analyze apoptosis of AMO-transfected hiPSCs induced by DNA damage, 1.5 × 10^6^ *TP53*-mutant hiPSCs were transfected with 20 µM of AMO and plated on matrigel coated 6-well plate. After electroporation, 2 d later, the transfected hiPSCs were pre-treated 0.2 µM of doxorubicin for 16 h. These cells were harvested and washed twice with pre-chilled PBS, and then resuspended in 100 µl of 1 × binding buffer at a concentration of 1 × 10^6^ cells/ml. Annexin V-FITC and Propidium Iodide double staining was performed in accordance with manufacturer’s protocol (Annexin V-FITC Apoptosis Detection Kit I, BD Pharmingen). Samples were acquired on a LSRFortessa X-20 cell analyzer (BD Bioscience), and the data were analyzed using FlowJo software (FlowJo, Ashland, OR, USA).

To compare the growth between the WT-, *TP53* mutants, AMO-transfected cells, the hiPSCs were each differentiated toward a myoblast using a Skeletal Muscle Differentiation Kit (amsbio). Differentiated cells were plated at a density of 2 × 10^5^ cells per well in a 4-well dish. Each myoblast was transfected with 20 µM AMO using Endoporter according to manufacturer’s instructions (Gene Tools). The myoblasts were pre-treated with 0.2 µM doxorubicin for 4 h. The medium was replaced by fresh medium without doxorubicin and maintained for 24 h. The cells were fixed using 4% paraformaldehyde (PFA, Biosesang) for 15 min at room temperature (RT), then washed three times with PBS. Permeabilization was performed using 0.3% Triton X-100 for 15 min, followed by blocking with 3% BSA in PBS for one hour at RT. Primary antibodies were diluted in PBS containing 1% BSA (Ki67 (1:1000), Abcam ab16667; p53 (1:200), Santa Cruz sc-126 and incubated overnight in a humidity staining chamber at 4 °C. After washing three times with PBS for 5 minutes each, cells were incubated with fluorescent secondary antibodies (Alexa Fluor 488 goat anti-mouse IgG (1:1000), Thermo Fisher A11001; and Alexa Fluor 594 goat anti-rabbit IgG (1:1000), Thermo Fisher A11012) diluted in PBS with 1% BSA for 1 h at RT. Cells were then washed twice in PBS and incubated with DAPI solution (1 µg/mL in PBS) for 10 min at RT. After a brief wash, cells were immersed in PBS. Images were captured using the EVOS M5000 imaging system (Thermo Fisher). Cells positive for Ki67 and DAPI were counted using the automated counting feature in the microscope’s equipped software.

### Statistical analysis

Statistical analyses were performed using GraphPad Prism version 8.0 (GraphPad Software, USA). Data are represented as column plots with mean ± SD. Differences between the two groups were assessed using an unpaired Student’s t-test. Comparisons among three groups were analyzed using one-way ANOVA or two-way ANOVA, depending on the experimental conditions. Multiple comparisons were conducted using Tukey’s post hoc test to correct for statistical significance. A p-value of < 0.05 was considered statistically significant. indicators: ∗*p* < 0.05, ∗∗*p* < 0.01, ∗∗∗*p* < 0.001, ∗∗∗∗*p* < 0.0001.

## Results

### Prediction of splicing mutation candidates from TP53 missense mutations

**Fig. 1:**
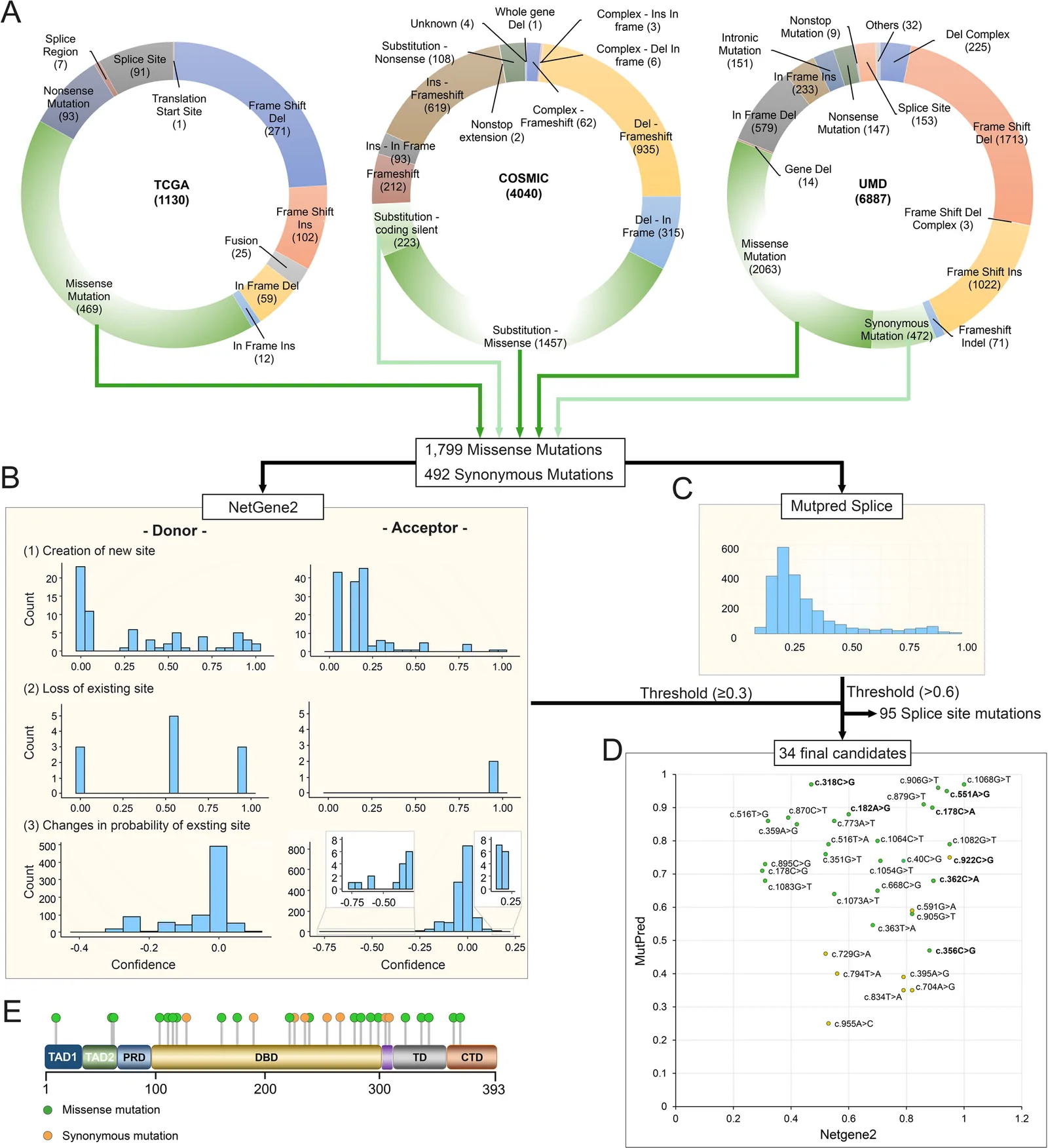
Selection of candidate *TP53* mutations affecting *TP53* splicing. **A ***TP53* mutations in Cancer Genome Database: The Cancer Genome Atlas (TCGA), Catalogue of Somatic Mutations in Cancer (COSMIC), and Universal Mutation Database (UMD). Number of *TP53* missense mutations or synonymous mutations isolated from Cancer Genome Database. **B**, **C ***TP53* splicing mutation candidates isolated by NetGene2 (**B**) or Mutpred (**C**) splice site predictor. Selection of candidates satisfying threshold values of 0.3 or higher (NetGene2) and 0.6 or higher (Mutpred) in relation to new splice site generation. Excluding 95 mutations occurring at splice sites. **D** Selection of 34 splicing mutation candidates by cross-referencing with NetGene2 selection, defaulting to NetGene2 results for candidates not identified by MutPred or below a threshold. Candidate mutations for minigene construction were indicated in bold style. Green dots, missense mutations; Orange dots, synonymous mutations. **E** Distribution of splicing mutation candidates at p53. TAD, Transactivation domain; DBD DNA-binding domain; TD, Tetramerization domain; CTD, C-terminal domain. Green lollipop, missense mutations; Orange lollipop, synonymous mutations

Among the various *TP53* mutation types (substitutions, insertions, and deletions), we primarily focused on single nucleotide substitutions to investigate their potential impact on splicing. To comprehensively analyze known *TP53* mutations, we integrated data from three major cancer mutation databases: The Cancer Genome Atlas (TCGA), Catalogue of Somatic Mutations in Cancer (COSMIC), and the Universal Mutation Database (UMD). From these databases, we retrieved 1130, 4040, and 6887 *TP53* mutations from TCGA, COSMIC, and UMD databases, respectively (Fig. 1A). Focusing on exonic single-nucleotide substitutions, we selected missense and synonymous mutations while excluding nonsense mutations that are typically predicted to result in loss of function. Missense mutations accounted for the largest proportion of mutations across all three databases. Specifically, 469 missense mutations were identified in TCGA. From COSMIC, 1457 “Substitution - Missense” and 223 “Substitution - coding silent” mutations were selected. From UMD, 2054 “Missense_Mutation” and 471 “Synonymous_Mutation” were included. After data integration, a total of 1,799 missense and 492 synonymous *TP53* mutations were compiled (Fig. 1A). To identify mutations with potential effects on splicing, we first applied NetGene2, a neural network–based splice-site prediction tool that assigns confidence scores (0–1) to sequence positions with potential splice-site activity [24]. NetGene2 was previously instrumental in predicting the novel splice site generated by the *TP53* c.359A > G mutation [8]. Based on the NetGene2 predictive score for this mutation (0.42), we set a conservative minimum confidence threshold of 0.3 to identify candidate mutations potentially associated with the creation of novel splice sites (Supplementary Table 1, left column). Among the 2,291 analyzed mutations, those exhibiting significant changes in predicted splice-site confidence were categorized into three groups: creation of new splice sites, loss of existing sites, and changes in the probability of existing sites (Fig. 1B). To further evaluate the likelihood that these variants could disrupt splicing, we additionally analyzed them using MutPred Splice, a machine learning–based prediction tool that estimates the probability of splicing disruption (Fig. 1C) [25]. Variants with MutPred scores above the default threshold (0.6) were considered likely to affect splicing, consistent with previous reports [26]. However, because our primary objective was to identify mutations predicted by NetGene2 to generate novel splice sites, variants predicted by NetGene2 were retained as candidates even when their MutPred scores were below this threshold (Supplementary Table 2). Using this approach, 34 mutations were predicted by NetGene2 to result in the creation of novel splice sites (Fig. 1D; Table 1). These included 26 mutations predicted to generate novel 5 splice sites (5’SS) and 8 predicted to generate novel 3’ splice sites (3’SS), which were distributed across the *TP53* gene (Fig. 1E). Among these 34 candidates, we prioritized missense mutations over synonymous mutations to investigate not only their potential impact on splicing but also the functional consequences of the resulting amino acid substitutions after splice-site restoration. 95 Mutations located at exonic positions flanking splice junctions were excluded from further analysis because these positions can directly affect splice-site recognition or disrupt exonic splicing regulatory elements, making splice disruption at these locations readily predictable.

**Table 1:** Prediction of splicing affected by 34 *TP53* mutations

TP53 Mutation				Splicing type		Confidence		Source		
CDS^a^	AA^b^	Type	exon	5´SS^c^	3´SS^c^	NetGene2	MutPred	TCGA	COSMIC	UMD
c.40 C > G	L14V	mis^d^	2	O		0.79	0.74			O
c.178 C > A	P60T	mis	4	O		0.89	0.90		O	O
c.178 C > G	P60A	mis	4	O		0.30	0.71		O	O
c.182 A > G	D61G	mis	4	O		0.60	0.88	O	O	O
c.318 C > G	S106R	mis	4	O		0.47	0.97	O	O	O
c.351G > T	G117G	syn^e^	4	O		0.52	0.76			O
c.356 C > G	A119G	mis	4	O		0.88	0.47		O	O
c.359 A > G	K120R	mis	4	O		0.42	0.85	O	O	O
c.362 C > A	S121Y	mis	4	O		0.90	-	O	O	
c.363T > A	S121S	syn	4	O		0.70	-		O	
c.395 A > G	K132R	mis	5		O	0.79	0.39	O	O	O
c.516T > A	V172V	syn	5	O		0.53	0.79		O	O
c.516T > G	V172V	syn	5	O		0.32	0.86		O	O
c.551 A > G	D184G	mis	5	O		0.94	0.95			O
c.591G > A	V197V	syn	6		O	0.82	0.59			O
c.668 C > G	P223R	mis	6	O		0.70	0.65		O	O
c.704 A > G	N235S	mis	7		O	0.82	0.35		O	O
c.729G > A	M243I	mis	7		O	0.52	0.46		O	O
c.773 A > T	E258V	mis	7	O		0.55	0.86		O	O
c.794T > A	L265Q	mis	8		O	0.56	0.40		O	O
c.834T > A	P278P	syn	8		O	0.79	0.35			O
c.870 C > T	R290R	syn	8	O		0.39	0.87		O	O
c.879G > T	G293G	syn	8	O		0.86	0.91			O
c.895 C > G	L299V	mis	8	O		0.31	0.73		O	O
c.905G > T	G302V	mis	8	O		0.82	0.58			O
c.906G > T	G302G	syn	8	O		0.91	0.96		O	O
c.922 C > G	L308V	mis	9		O	0.95	0.75		O	O
c.955 A > C	K319Q	mis	9		O	0.53	0.25			O
c.1054G > T	D352Y	mis	10	O		0.71	0.74			O
c.1064 C > T	A355V	mis	10	O		0.70	0.80		O	O
c.1068G > T	G356G	syn	10	O		1.00	0.97		O	O
c.1073 A > T	E358V	mis	10	O		0.55	0.64		O	O
c.1082G > T	G361V	mis	10	O		0.95	0.79			O
c.1083G > T	G361G	syn	10	O		0.31	0.68		O	O

^a^CDS, coding DNA sequence; ^b^AA, amino acid; ^c^SS, Splice Site; ^d^mis, missense; ^e^syn, synonymous

### Evaluation of missense mutations affecting constitutive splicing

**Fig. 2:**
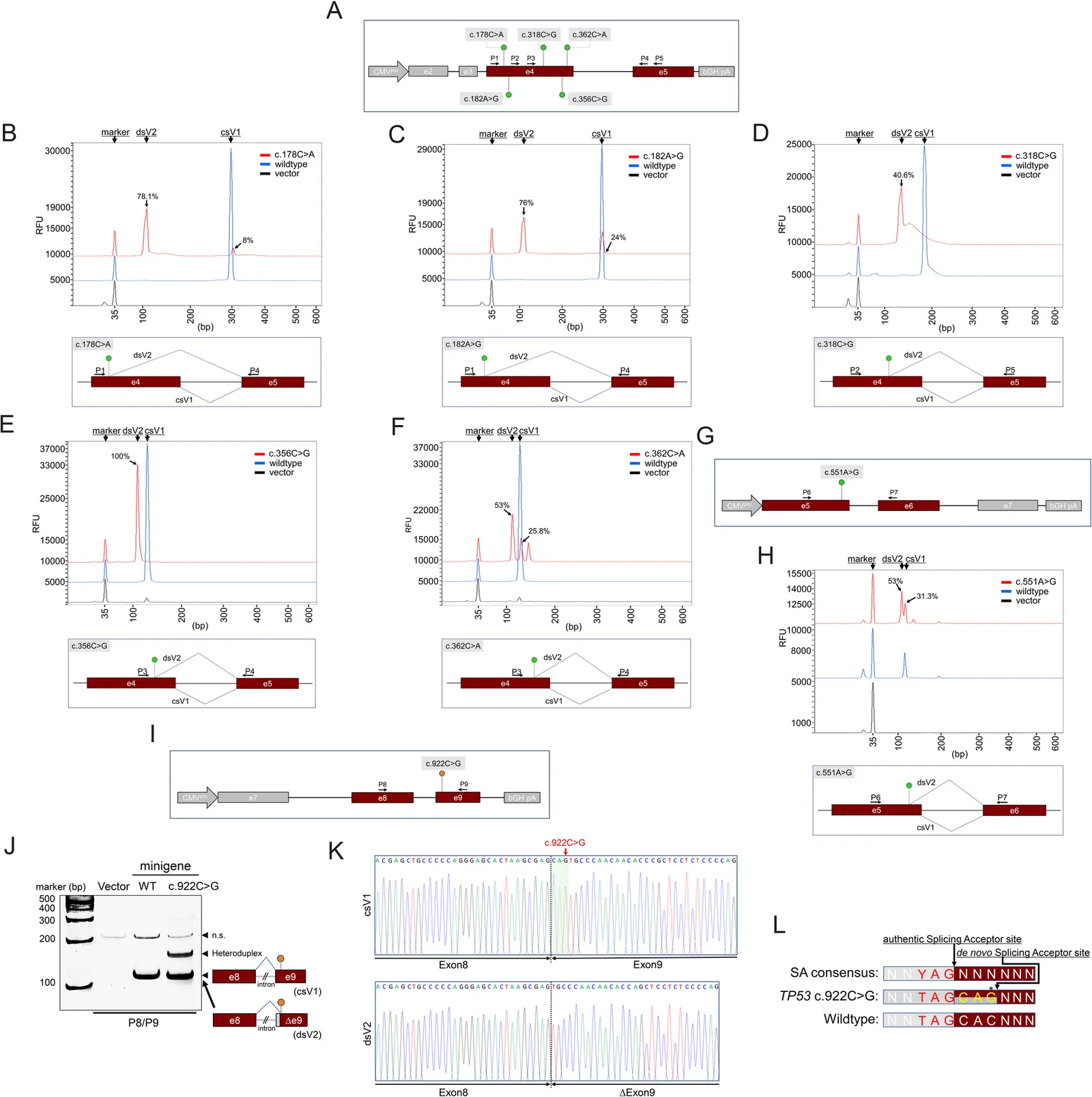
Evaluation of *TP53* splicing mutation candidates. **A** Minigene constructs of c.178 C > A, c.182 A > G, c.318 C > G, c.356 C > G, and c.362 C > A mutations and locations of primer pairs to confirm splicing alterations caused by these mutations. **B**–**F** Fragments analyzed from the minigene construct of c.178 C > A mutation (**B**), c.182 A > G mutation (**C**), c.318 C > G mutation (**D**), c.356 C > G mutation (**E**), c.362 C > A mutation (**F**). **G** Minigene construct of c.551 A > G mutation and primer pairs to confirm splicing alterations caused by the mutation. **H** Fragments analyzed from the minigene construct of c.551 A > G mutation. **I** Minigene construct of c.922 C > G mutation and primer pairs to confirm splicing alterations caused by the mutation. **J** Fragments analyzed from the minigene construct of c.922 C > G mutation on a 10% DNA polyacrylamide gel. **K** DNA sequencing analysis of the fragments. **L** Comparison between canonical splicing acceptor and novel splicing acceptor created by c.922 C > G mutation. CMV^pro^, Cytomegalovirus promoter; csV1, constitutive splicing Variant 1; dsV2, *de novo*-splicing Variant 2. See also Supplementary Fig. 1

To test our hypothesis that selected missense mutations authentically generate novel splice sites, we constructed minigene systems encompassing multiple exons and introns centered around each mutation site (Fig. 2A, G, and I). Candidate mutations for minigene construction were randomly selected from exons encoding the proline-rich or DNA binding domains, including c.178 C > A, c.182 A > G, c.318 C > G, c.356 C > G, c.362 C > A, c.551 A > G, and c.922 C > G (Fig. 1D, bold style). Each minigene was transfected into H1299 cancer cells, lacking endogenous *TP53*, to evaluate the presence of alternative transcript variants and quantify the proportion of constitutively spliced transcripts. A recent study by Fortuno et al. systematically evaluated predicted splice-altering *TP53* variants using a minigene assay and reported that several exonic variants can induce aberrant splicing events [27]. Our results provide independent experimental confirmation of splicing disruption for several variants examined in that study. For the c.178 C > A mutation, located within the proline-rich domain, an alternative transcript (designated *de novo*-splicing variant 2, dsV2) was predominantly expressed, accounting for 78.1% of the *TP53* transcript. In contrast, the constitutive splicing variant 1 (csV1) accounted for only approximately 8% of total transcripts (Fig. 2B; Supplementary Fig. 1A, left). Sequence analysis of dsV2 revealed that part of exon 4, including the sequence immediately downstream of the mutation site, was skipped and instead directly spliced to exon 5 (Supplementary Fig. 1A, middle). This suggests that previously non-splice-related sequences were reorganized into a functional splice donor site as a result of the mutation (Supplementary Fig. 1A, right). Notably, aberrant splicing associated with the c.178 C > A variant was also reported in the minigene study by Fortuno et al., supporting the interpretation that this variant activates a cryptic splice donor site [27]. Similarly, the c.182 A > G mutation, located in the proline-rich domain, strongly promoted dsV2 formation. In this case, dsV2 represented 76% of the *TP53* transcript, while csV1 constituted only 24% (Fig. 2C; Supplementary Fig. 1B, left). Sequence analysis showed that the mutation was located precisely at the boundary between exon and intron sequences, suggesting that it plays a role in generating a novel splice donor site (Supplementary Fig. 1B, middle and right). Consistent with our observations, Fortuno et al. also reported substantial splice disruption associated with this variant [27]. While both c.178 C > A and c.182 A > G significantly reduced csV1 expression, the c.318 C > G and c.356 C > G mutations completely abolished csV1 expression, producing only dsV2 (Fig. 2D, E; Supplementary Fig. 1C, D). This indicates that the newly formed splice sites generated by the c.318 C > G or c.356 C > G mutations outcompeted the constitutive splice sites, resulting in a complete shift in splicing preference (Supplementary Fig. 1C, D). These findings are in agreement with the systematic minigene analysis by Fortuno et al., which similarly identified strong cryptic splice-site activation for these variants [27]. The c.362 C > A mutation in exon 4 and c.551 A > G mutation in exon 5 also created a novel 5’SS that generates dsV2, which accounts for 53% of *TP53* transcripts in both cases (Fig. 2F, H; Supplementary Fig. 1E, F). Due to the frequent splicing events in this novel 5’SS site, csV1 was detected at reduced levels, accounting for only 25.8% and 31.3% of transcripts, respectively (Fig. 2F, H). Similar splice-disruptive effects for these variants were also reported in the previous *TP53* minigene study, further supporting the robustness of these observations [27]. While these mutations were commonly associated with the creation of novel 5’SS, the c.922 C > G mutation, located immediately downstream of the canonical splicing acceptor site in exon 9, directly induced the formation of a novel 3’SS (Fig. 2I, J). Although the 2-bp gap between the authentic and novel 3’SS posed a challenge for quantitative fragment analysis, we clearly demonstrated the presence of transcripts generated from the novel 3’SS (Fig. 2K, L). Similar splice alterations associated with this variant have also been noted in previous functional analyses of *TP53* variants [27]. Overall, the minigene assay qualitatively revealed cryptic splicing events driven by specific missense mutations, consistent with predictions from computational splicing tools and with previous systematic minigene studies of *TP53* variants [27]. To further investigate their phenotypic consequences, we attempted to create genetically modified mutant cell lines.

### Generation of TP53-mutant knock-in cells

We developed homozygous knock-in cell lines harboring the c.182 A > G, c.318 C > G, c.356 C > G, and c.362 C > A mutations using bacterial artificial chromosomal DNA-mediated homologous recombination (Supplementary Fig. 2A), a method previously established for the generation of c.359 A > G mutant cells [8, 28]. Genetic modifications of the knock-in cells were confirmed through genotyping and sequence analysis (Supplementary Fig. 2B). The selection of candidate mutations for each knock-in cell line was based on the degree of residual csV1 expression, which was assessed according to the results from the minigene assay, in the following order: c.362 C > G, c.182 A > G, c.356 C > G, and c.318 C > G. First, we examined the expression pattern of p53 protein in each *TP53*-mutant knock-in cell line. Under genotoxic stress induced by doxorubicin treatment, the expression of mutant p53 protein encoded by the c.182 A > G mutation was relatively lower than that of the wildtype p53 protein (Supplementary Fig. 2C). More strikingly, the c.318 C > G or c.356 C > G mutations resulted in mutant p53 proteins that were barely detectable (Supplementary Fig. 2C). The low expression of these mutant p53 proteins, except for c.362 C > A (which showed similar levels to wildtype p53), was directly reflected in the expression of p21 and PUMA, which are representative p53 target genes (Supplementary Fig. 2C). In addition, the mRNA expression levels of Cyclin Dependent Kinase Inhibitor 1 A (*CDKN1A*) and BCL2 Binding Component 3 (*BBC3*) genes, encoding the corresponding proteins, also mirrored the protein levels (Supplementary Fig. 2D).

While the reduced expression of p53 protein can be attributed to the loss of p53 stabilization, which typically increases under genotoxic stress, it may also be related to the expression level of mutant *TP53* mRNA, specifically csV1. Indeed, the quantitative pattern of protein expression induced by *TP53* mutations was correlated with the level of *TP53* csV1 expression. Compared to wildtype *TP53* mRNA expression, the levels of mutant *TP53* csV1 were less than 10% in both c.318 C > G and c.356 C > G mutant cells and approximately 25% in c.182 A > G mutant cells (Supplementary Fig. 2D). In contrast, the c.362 C > A mutation resulted in high *TP53* csV1 expression, almost equivalent to wildtype *TP53* expression (Supplementary Fig. 2D). Therefore, p53 protein abundance was determined more by the level of mutant *TP53* csV1 rather than by protein instability.

In general, the copy number of a transcript is affected by both expression efficiency and mRNA stability, which are regulated at the transcriptional and post-transcriptional levels [29]. Given that splicing changes due to mutations affect transcript abundance, as demonstrated by minigene assays, we aimed to quantitatively investigate *TP53* mRNA levels in mutant cells from a splicing perspective to elucidate the mechanistic basis underlying these expression patterns.

### Splicing modulation of novel 5’ splice sites generated by TP53 mutations

**Fig. 3:**
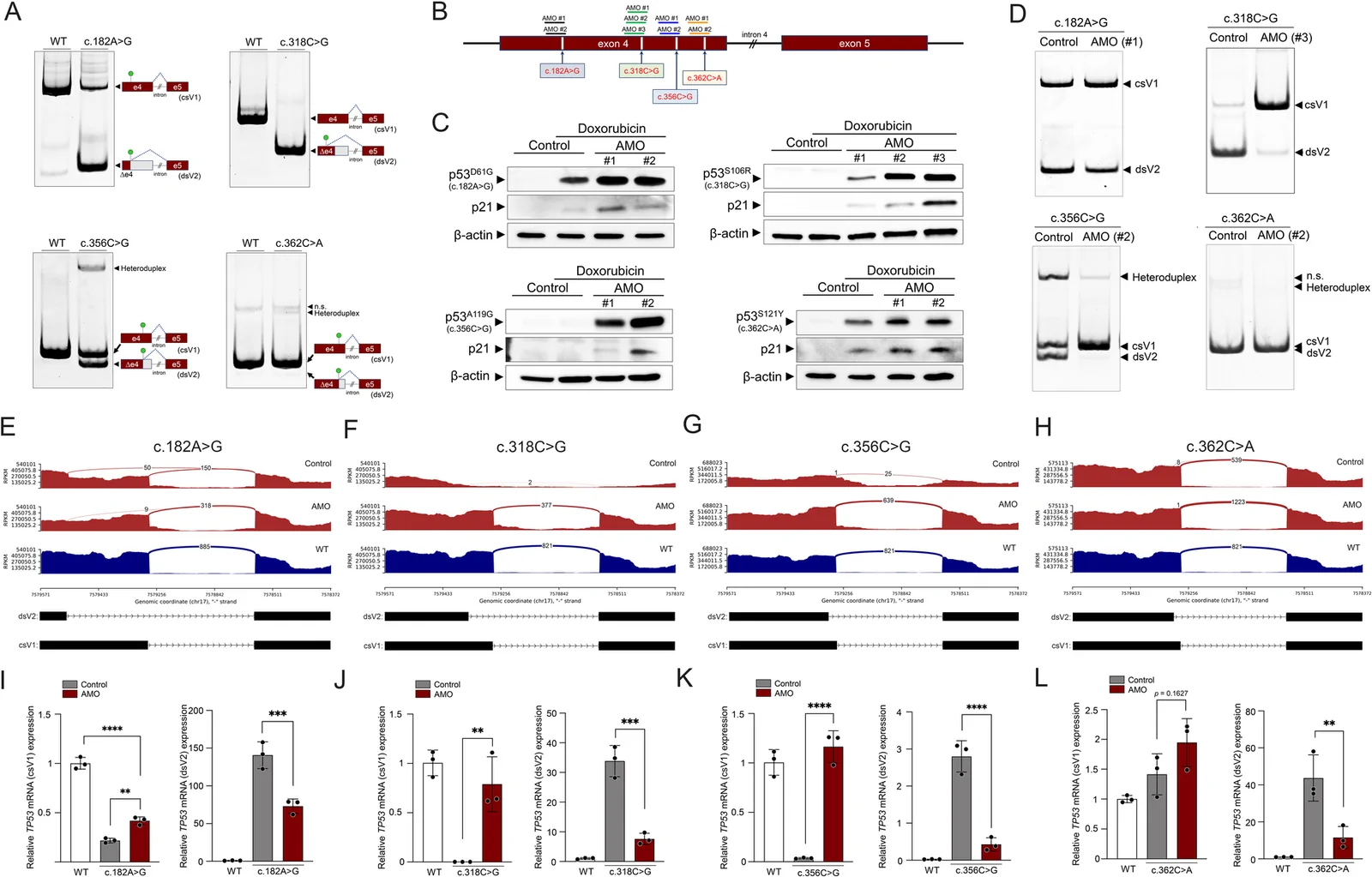
Splicing Modulation of novel 5’ splice sites generated by *TP53* mutations. **A** Novel 5’ splice sites generated in the *TP53*-mutant cells, including c.182A > G, c.318C > G, c.356C > G, and c.362C > A mutations. **B** AMOs masking the novel 5’ splice site generated by *TP53* mutations, respectively. **C** AMOs that effectively mask novel 5’ splice site. **D** Evaluation of effective AMOs by fragment analysis on 10% DNA polyacrylamide gels. **E**–**H** Sashimi plots generated from RNA-seq reads across exon 4–5 with and without AMO treatment. c.182 A > G (**E**), c.318 C > G (**F**), c.356 C > G (**G**), c.362 C > A (**H**), respectively. **I**–**L** Real-time PCR to measure the expression of csV1 or dsV2 under 20 µM AMO treatment for 24 h. c.182 A > G (**I**), c.318 C > G (**J**), c.356 C > G (**K**), c.362 C > A (**L**), respectively. All reactions were performed in technical triplicates to ensure reproducibility. Data are represented as column plots with mean ± SD. Differences between the two groups were assessed using an unpaired Student’s t-test, ^**^p < 0.01, ^***^p < 0.001, ^****^p < 0.0001. AMO, Antisense Morpholino Oligomer

In *TP53*-mutant knock-in cells, both csV1 and dsV2 transcripts were qualitatively detected. For the c.182 A > G and c.318 C > G mutations, the expression patterns of these transcripts were consistent with the results from the minigene assay (Fig. 3A). However, in c.356 C > G mutant cells, csV1 was more abundant than dsV2; however, the copy number of csV1 remained low and was therefore considered negligible. (Fig. 3A, G, control). In contrast, the c.362 C > A mutation, which was expected to show leaky csV1 expression, exhibited minimal dsV2 production, with csV1 accounting for almost all transcript expression (Fig. 3A, H, control).

To further investigate the functional role of mutant sequences in splicing modulation and expression dynamics, we employed antisense morpholino oligomers (AMOs). These AMOs are designed to target specific pre-mRNA regions, enabling them to selectively bind to *de novo*-splice sites and block aberrant splicing events. This interference promotes the use of constitutive splice donor sites, thereby increasing csV1 expression [9, 30–32]. Among AMOs specifically designed to complement mutant sequences, we identified optimal candidates capable of enhancing p53 protein expression by masking the novel 5’SS splice sites (Fig. 3B, C). Indeed, the optimal AMOs successfully blocked the novel 5’SS, restoring the constitutive splicing pattern, as demonstrated by the recovery of csV1 expression (Fig. 3D). In c.182 A > G mutant cells, AMO treatment doubled csV1 expression while dramatically reducing dsV2 levels (Fig. 3E, I). Similarly, in c.318 C > G and c.356 C > G mutant cells, csV1 expression markedly increased, while dsV2 reached near-undetectable levels (Fig. 3F, G, J, and K). For the c.362 C > A mutation, which initially showed minimal splicing disruption, AMO treatment yielded modest effects, further reducing the scarce dsV2 levels (Fig. 3H, L).

We extended our analysis to cancer cell lines harboring mutations that affect splicing (Supplementary Fig. 4A, F). The frequently observed c.318 C > G mutation, found in liver or oral cavity cancers [33–36], was characterized in SNU-368 hepatocellular carcinoma cells, where we confirmed the formation of a novel 5’SS splice site (Supplementary Fig. 4B). AMO treatment in these cells successfully restored csV1 expression and increased p53 protein levels, recapitulating the results observed in knock-in models (Supplementary Fig. 4C, D, and E). In the SKN-3 oral cavity squamous cell carcinoma cell line, restriction fragment length polymorphism analysis revealed a heterozygous genotype for both the c.318 C > G and c.461_462GC > TT mutations (Supplementary Fig. 4F, G). Based on our findings, the baseline mutant p53 protein in this cell line was likely expressed from the allele harboring the c.461_462GC > TT mutation, as the protein derived from the c.318 C > G allele was presumably absent due to splicing defects (Supplementary Fig. 4H). Following AMO treatment, mutant p53 and its downstream target protein levels significantly increased, likely as a result of effective masking of the novel 5’SS site introduced by c.318 C > G mutation (Supplementary Fig. 4H). These findings confirmed that the aberrant splicing caused by the c.318 C > G mutation underlies the defective p53 expression observed in these cancer cell lines and that AMO-based splicing correction can restore functional p53 expression.

#### Splicing modulation by U1 small nuclear RNA and stability of de novo-splicing variant 2

In addition to AMOs, we explored the potential role of U1 small nuclear RNA (snRNA) in enhancing constitutive splicing. As a key component of the spliceosomal U1 snRNA, U1 snRNA recognizes 5’SS in pre-mRNA, initiating spliceosome assembly and facilitating accurate splicing [37–39]. This recognition occurs via complementary base pairing between the 5’ nine-nucleotide sequence of U1 snRNA and the 5’SS sequence of the target pre-mRNA (Supplementary Fig. 3B). While U1 snRNA typically binds complementarily to exon-intron junctions, it may also recognize novel 5’SS generated by *TP53* mutations. To preferentially reinforce recognition of the constitutive splice site sequence located at the exon 4-intron 4 boundary of *TP53*, we engineered a modified U1 snRNA (U1^mut^) that harbored containing a canonical 5’SS complementary sequence (Supplementary Fig. 3A, B) [40]. We hypothesized that U1^mut^ expression would shift splicing from mutation-driven alternative events to constitutive splicing. Despite this targeted enhancement, only c.318 C > G mutant cells showed modest csV1 recovery. No changes were detected in other mutant cell lines, suggesting that wildtype U1 snRNA competed effectively at most sites, limiting the impact of U1^mut^ (Supplementary Fig. 3C). Notably, the selective effect observed in c.318 C > G mutant cells likely reflected inherently weaker recognition of the constitutive splice site by wildtype U1 snRNA, allowing U1^mut^ snRNA to exert a modest compensatory effect (Supplementary Fig. 3C, D). However, the overall impact on p53 protein function remained minimal (Supplementary Fig. 3F), and the reciprocal expression dynamics between dsV2 and csV1 were largely unaffected (Supplementary Fig. 3D, E).

We also investigated the quantitative aspects of dsV2 transcripts generated from novel 5’SSs. Splicing frequency and transcript stability influenced dsV2 expression. As constitutive and alternative splicing events are reciprocal, dsV2 levels should be considered alongside csV1 expression. Expression levels of dsV2 expressed at mutation sites, including c.318 C > G, c.356 C > G, and c.362 C > A mutations, excluding the c.182 A > G mutation, were extremely low (Fig. 3E-H, control). Importantly, the reasons for low dsV2 levels varied among mutations. In c.362 C > A mutant cells, csV1 expression was robust and comparable to wildtype *TP53*, suggesting that strong constitutive splicing outcompeted aberrant alternative splicing events, minimizing dsV2 expression (Fig. 3H, L). This low dsV2 abundance was therefore attributed to splicing dynamics, not transcript instability. In contrast, both csV1 and dsV2 expression were significantly low in c.318 C > G and c.356 C > G mutant cells (Fig. 3F, G, J, and K), suggesting an alternative mechanism of transcript depletion. We hypothesized frameshift mutations introduced by exon skipping may generate premature termination codons (PTCs), leading to degeneration of dsV2 transcripts via the nonsense-mediated mRNA decay (NMD) pathway, an RNA surveillance mechanism [41, 42]. To test this hypothesis, we temporarily suppressed NMD by silencing UPF1, a key NMD component [43], using short interfering RNA (siUPF1), and monitored dsV2 levels. Following siUPF1 treatment, dsV2 levels in c.318 C > G and c.356 C > G mutant cells increased significantly, by 3.5-fold and 1.7-fold, respectively, implicating NMD in their degradation (Supplementary Fig. 3H, I). Conversely, c.182 A > G mutant cells exhibited high baseline dsV2 levels and showed little quantitative change upon NMD inhibition, indicating that dsV2 is both splicing-efficient and stable (Supplementary Fig. 3G). In c.362 C > A mutant cells, the persistently low dsV2 levels remained unaffected by UPF1 silencing, supporting the notion that limited splicing, not degradation, was responsible for dsV2 scarcity (Supplementary Fig. 3J).

### Comprehensive analysis to identify restoration of AMO-mediated restoration of the TP53 cellular pathway

**Fig. 4:**
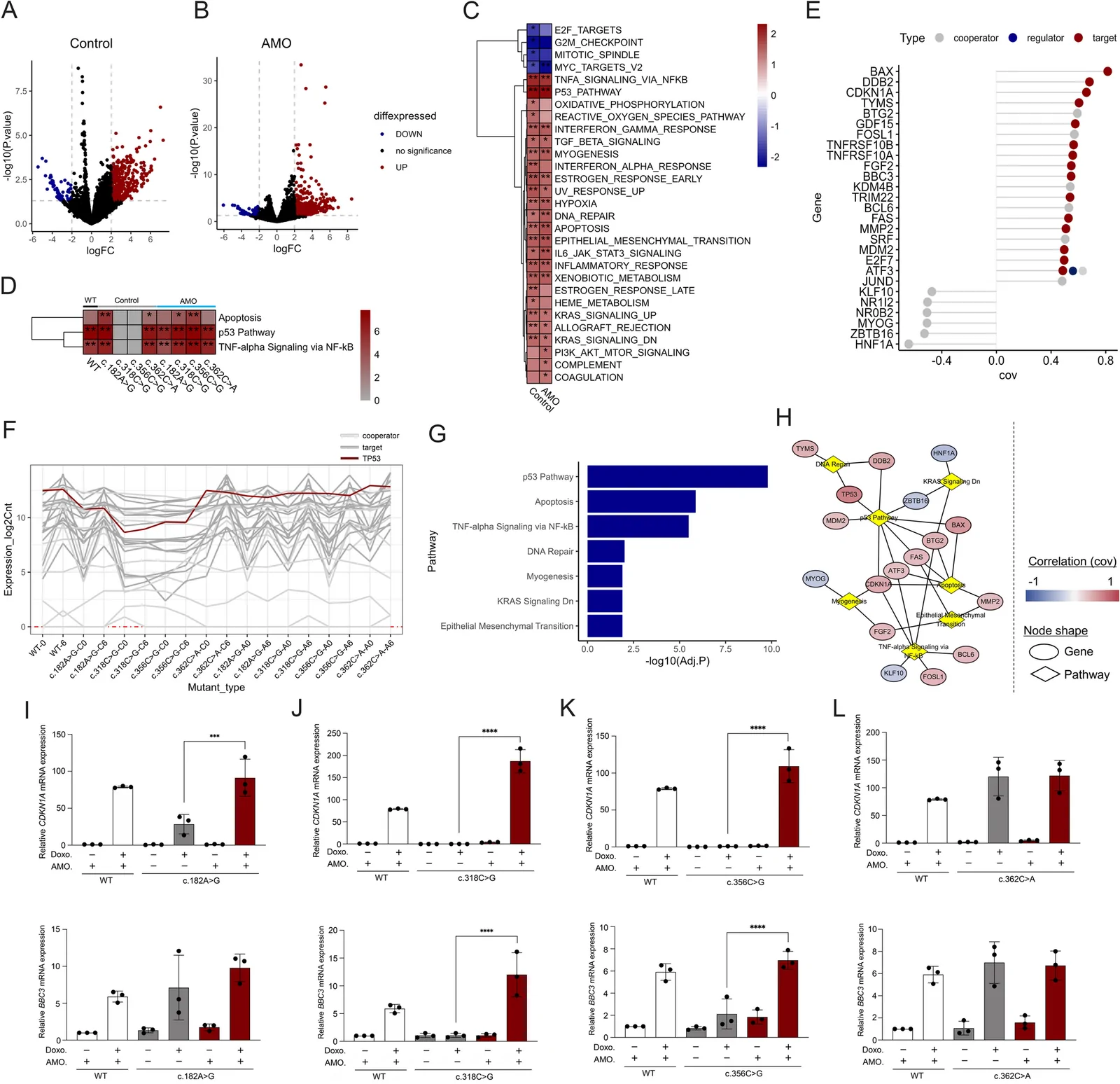
Comprehensive analysis to identify AMO-mediated *TP53* cellular pathway. **A**, **B** Volcano plot showing differentially expressed genes (DEGs) between doxorubicin-treated and -untreated Control groups (**A**), and between doxorubicin-treated and -untreated AMO groups (**B**). Red and blue dots represent significantly upregulated and downregulated genes, respectively (|logFC| > 2 and *p* < 0.05). **C** A heat map of gene set enrichment results for AMO-treated (AMO) and -untreated *TP53* mutations groups (Control) using Hallmark gene set. *p < 0.05 and **p < 0.01. **D** Heatmap showing common molecular mechanisms associated with each *TP53* mutation. ^*^p < 0.05 and ^**^p < 0.01. **E** Target genes co-expressed with *TP53* mutations identified by correlation analysis. The x-axis represents the correlation coefficient between *TP53* mutations and target gene expression, while the y-axis shows the list of significantly co-expressed target genes (*p* < 0.05). **F** Expression patterns of *TP53* and its co-expressed target genes. The red line represents *TP53* expression levels, while the gray lines represent the expression values of co-expressed target genes. **G** Enriched functional pathways associated with genes co-expressed with *TP53* mutations. The x-axis represents the significance of functional terms. **H** Integrated network of functional pathways and significantly co-expressed target genes. Node colors indicate correlation coefficient values between *TP53* mutations and target gene expression. **I**–**L** Real-time PCR to validate the expression of p53 representative target genes, *CDKN1A* or *BBC3*, c.182 A > G (**I**), c.318 C > G (**J**), c.356 C > G (**K**), c.362 C > A (**L**), respectively. All reactions were performed in technical triplicates to ensure reproducibility. Data are represented as column plots with mean ± SD. Differences between the two groups were assessed using an unpaired Student’s t-test, ***p < 0.001, ****p < 0.0001. AMO, Antisense Morpholino Oligomer

Mutations initially classified as hypomorphic can potentially be converted into functional missense mutations via AMO treatment. To explore the molecular effects of this conversion, we analyzed gene expression profiles in four *TP53* mutant cell lines before and after AMO treatment. Cells were treated with either AMO or control oligonucleotides and subsequently exposed to either doxorubicin or vehicle (DMSO). RNA-seq analysis primarily focused on comparing AMO-treated and control cells under doxorubicin treatment to assess the effect of AMO on DNA damage–induced p53-dependent transcription. In both groups, across all mutants, AMO treatment resulted in a higher proportion of upregulated genes than downregulated genes, indicating a broad activation of gene expression (Fig. 4A, B; Supplementary Table 3). To identify the biological pathways influenced by AMO, we performed gene set enrichment analysis (GSEA). In c.318 C > G and c.356 C > G mutation cells, several key pathways, including “Apoptosis,” “p53 pathway,” and “TNF-alpha signaling via NF-kB,” were significantly enriched following AMO treatment (Fig. 4C, D). These findings suggest successful reactivation of p53-dependent transcriptional programs in these two mutants. In contrast, enrichment for the “Apoptosis” pathway diminished in the AMO-treated c.182 A > G and c.362 C > A cells, indicating mutation-specific variability in AMO responsiveness. These results suggest that AMO treatment was more effective in restoring *TP53* expression and function in the c.318 C > G and c.356 C > G mutants, which exhibited minimal *TP53* expression before treatment. We hypothesized that the AMO-mediated recovery of *TP53* expression led to the reactivation of downstream target genes, restoring core p53 molecular functions. To explore this hypothesis, we performed a correlation analysis between *TP53* expression and its target genes using the TRRUST database. After AMO treatment, 77.8% of the identified target genes were positively correlated with *TP53* expression, while 22.2% were negatively correlated (Fig. 4E; Supplementary Table 4). Notably, canonical p53 target genes, including *BAX*, *BBC3*, and *CDKN1A*, were significantly positively correlated with AMO-treated *TP53* mutants, whereas p53 cooperators, such as *KLF10* and *HNF1A*, showed a negative correlation. We further examined the target gene expression across the four *TP53* mutants. In c.318 C > G and c.356 C > G cells, *TP53* expression was almost undetectable in the absence of AMO but was restored to near wildtype *TP53* levels. In contrast, the c.182 A > G and c.362 C > A mutants retained baseline *TP53* expression comparable to those of the wildtype even without AMO, suggesting partial preservation of *TP53* function. Nevertheless, AMO treatment further enhanced p53 target gene expression in these mutants (Fig. 4F). Functional enrichment analysis of genes co-expressed with AMO-treated *TP53* revealed significant associations with key p53-regulated pathways, including the “p53 pathway (Adjusted P-value: 1.61 × 10^− 10^)”, “Apoptosis (Adjusted P-value:1.33 × 10^− 6^)”, and “DNA repair (Adjusted P-value:9.7 × 10^− 3^)” (Fig. 4G). An integrated molecular network illustrating these enriched pathways and their associated target genes is shown in Fig. 4H. These results suggest that AMO-mediated recovery of *TP53* expression reactivates crucial p53-related pathways.

Finally, we validated the restoration of p53 function by evaluating the expression of *CDKN1A* and *BBC3*, key target genes related to the role of p53 in cell growth inhibition and apoptosis. In the c.182 A > G mutant, *BBC3* expression remained close to normal levels despite low p53 protein expression, while *CDKN1A* remained unchanged (Fig. 4I). AMO treatment significantly increased p53 protein levels, which in turn enhanced *CDKN1A* expression (Fig. 4I). In the c.318 C > G and c.356 C > G mutants, p53 expression was undetectable without AMO; however, treatment fully restored both p53 expression and downstream target gene activation (Fig. 4J, K). In contrast, the c.362 C > A mutant maintained relatively normal p53 protein levels and target gene expression even without AMO (Fig. 4L). Collectively, these results indicate that p53 protein abundance, rather than the specific amino acid changes, is the primary determinant of transcriptional regulation in *TP53* mutants. The observed restoration of key target genes, particularly those involved in the p53 pathway, suggests that p53 function was effectively restored by AMO treatment. These findings suggests that AMO treatment has the potential to restore the function of p53 in *TP53* mutants, reactivating critical molecular pathways, such as apoptosis and DNA repair, which are crucial for cellular homeostasis.

#### Restoration of p53 function by AMO treatment in TP53 mutants

**Fig. 5:**
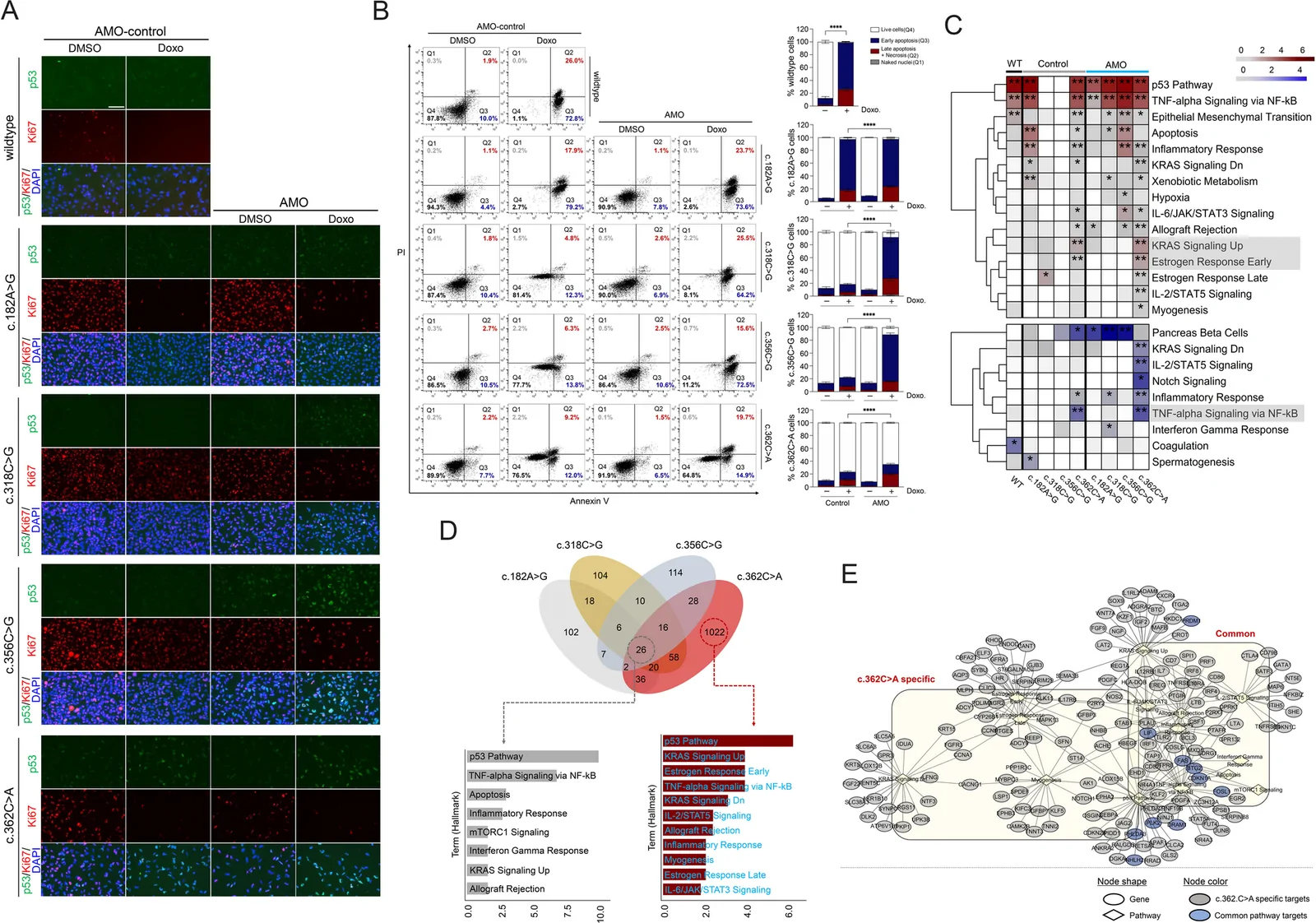
Restoration effect of p53 function by AMO. **A** Cell growth arrest analysis in the myoblasts derived from WT- and *TP53*-mutant hiPSCs. These cells, transfected with AMOs, were either untreated or treated with 0.2 µM doxorubicin for 12 h. The growth of differentiated cells was assessed by identifying cells stained with Ki67 (red). The p53 protein and nuclei were stained with α-p53 (green) and DAPI (blue), respectively. Scale bar = 100 μm. **B** Apoptosis in WT- and *TP53*-mutant hiPSCs was analyzed with or without 0.2 µM doxorubicin treatment for 16 h after AMO transfection. The apoptotic cells were assayed by staining with Annexin V-FITC for FACS analysis. Mean values from three independent experiments are shown with the standard derivation. The data represent mean ± SD from three independent experiments. ****p < 0.0001. AMO, Antisense Morpholino Oligomer. **C** Heatmap of key molecular features before and after doxorubicin treatment in wildtype (WT), four types of *TP53* mutations (Control), and four types of AMO-treated *TP53* mutations. *p < 0.05 and **p < 0.01. The upper and lower panels show functional analysis results for significantly up-regulated and down-regulated genes, respectively. The color scale represents the combined score (CS) provided by enrichR. **D** Comparison of differentially expressed genes (DEGs) and their molecular functions among the four types of AMO-treated *TP53* mutations. The Venn diagram in the top panel shows the number of DEGs in each doxorubicin-treated *TP53* mutation compared to -untreated mutations in the AMO groups, highlighting both commonly shared DEGs (common DEGs) and c.362 C > A -specific DEGs. The bottom panel presents enriched molecular pathways associated with the highlighted DEGs, showing significantly enriched molecular pathways that regulate both common and c.362 C > A -specific DEGs. **E** Integrated network of key mechanisms and target genes in shared targets (common) and c.362 C > A-specific targets. Yellow squares represent molecular pathways and target genes that are either common or specific to the c.362 C > A mutation

Pluripotent stem cells are highly sensitive to genotoxic stress-induced cell death, whereas differentiated myoblasts are resistant to cell death, but exhibit growth inhibition. To assess the effect of mutant p53 proteins on cell growth inhibition, we induced myoblast differentiation in *TP53*-mutant stem cells. Under genotoxic stress, AMO-treated c.182 A > G mutant cells did not significantly affect cell growth. This may be due to subtle differences in *CDKN1A* target gene expression or the degree of p53 pathway activation (Fig. 5A). In contrast, the c.318 C > G and c.356 C > G mutants, initially deficient in p53-mediated responses, showed no cell growth inhibition in the absence of p53 deficiency; however, AMO-mediated p53 restoration significantly reduced growth (Fig. 5A), likely due to the activation of the p53 pathway and *CDKN1A* expression. In contrast, the c.362 C > A mutant cells exhibited consistent growth inhibition regardless of AMO treatment, with stable *CDKN1A* expression, indicating limited responsiveness to splicing correction (Fig. 5A).

Next, we examined the pro-apoptotic function of p53, particularly via *BBC3*, a pro-apoptotic gene, to determine the impact of AMO treatment on p53-driven cell death. In c.182A > G mutant cells, AMO treatment increased p53 protein levels. However, under doxorubicin treatment, *BBC3* expression did not show a noticeable increase compared with AMO control cells treated with doxorubicin. Consistent with this observation, the total apoptotic cell population, including early apoptotic cells, remained comparable between the AMO-treated and AMO control groups. Interestingly, a slight increase in the proportion of late apoptotic cells was observed following AMO treatment. (Fig. 5B). For c.318C > G and c.356C > G mutants, cell death was almost completely lost due to p53 deficiency, but AMO treatment restored p53 protein levels and apoptosis (Fig. 5B). Interestingly, in c.362C > A mutants, more than half of the cells survived despite the restoration of p53 expression, suggesting that survival signals can interfere with p53-mediated apoptosis (Fig. 5B). Consistent with these observations, the c.362C > A variant retained relatively high levels of *TP53* expression and p53 target gene activity even in the absence of AMO treatment. This finding suggests that the cryptic splice donor generated by this mutation has limited strength, while the authentic splice donor site remains predominantly utilized. As a result, the major transcript produced through authentic splicing is maintained at a relatively high baseline level, which may reduce the magnitude of the functional effect induced by AMO-mediated splicing correction. To further investigate the molecular basis of this survival phenotype in the c.362C > A mutant, we performed GSEA on gene expression profiles before and after AMO treatment. Surprisingly, pathways such as “KRAS signaling Up,” “Estrogen Response Early,” and “TNF-α signaling via NF-kB” were significantly upregulated in both control and AMO-treated c.362C > A mutants (Fig. 5C). To further examine the characteristics of the AMO-treated c.362C > A mutant, we analyzed differentially expressed genes (DEGs) among the four AMO-treated *TP53* mutants using a Venn diagram (Fig. 5D; Supplementary Table 5). We focused on two DEG subsets: [1] common DEGs shared among all four *TP53* mutants, and [2] c.362C > A-specific DEGs. Functional enrichment analysis revealed that the common DEGs were significantly enriched in 8 functional pathways, whereas 11 functional pathways were significantly enriched in the c.362C > A-specific group (Fig. 5D). To compare the significantly enriched functional pathways and their associated target genes between the two groups, we constructed a pathway-target network (Fig. 5E). In the common group, the pathway with the most shared targets was the “p53 pathway,” which included *FAS*, *BTG2*, *CDKN1A*, *DRAM1*, *PLK2*, *RHLDA3*, and *NHLH2*. In the c.362C > A-specific group, the enriched pathways included “KRAS Signaling Dn,” “Estrogen Response Early,” “Estrogen Response Late,” and “Myogenesis.” These findings suggest that, unlike the other *TP53* mutants analyzed, c.362 C > A mutant cells retain typical p53 pathway activity while also exhibiting additional signaling pathway signatures associated with cellular transformation. Notably, these pathway signatures were observed both before and after AMO treatment, indicating that they likely represent mutation-specific transcriptional characteristics rather than direct consequences of splicing correction.

## Discussion

In this study, we identified several mutations previously classified as missense mutations that exhibited characteristics of hypomorphic *TP53* mutations. These mutations reduce or disrupt *TP53* transcript levels caused by mutation-mediated splicing aberrations rather than amino acid substitutions. Notably, the mutations we characterized primarily created alternative 5’SS or 3’SS, rather than causing splicing events, such as exon skipping, mutually exclusive exons, or intron retention. Among these, the c.362 C > A mutation creates a novel 5’SS identical to that produced by the previously characterized c.359 A > G mutation, indicating coexistence of two mutations within the same splice site region [8].

Despite these similarities, the effect of each mutation on constitutive splicing differs markedly: c.359 A > G results in negligible csV1 expression, whereas the c.362 C > A allows for abundant csV1 expression. This disparity suggests that the novel c.359 A > G mutation-generated 5’SS exerts a stronger suppressive effect on the authentic splice site than that generated by c.362 C > A. Interestingly, while both mutations lead to frameshift-induced dsV2 variants containing PTC; only the dsV2 variant from the c.359 A > G mutation is targeted by NMD. In contrast, the dsV2 from c.362 C > A escapes NMD targeting.

Given that constitutive and alternative splicing events are reciprocal, it indicates that the predominance of csV1 in c.362 C > A mutants naturally limits the frequency of alternative splicing at the novel 5’SS. Similarly, the c.318 C > G mutation generates a dsV2 transcript that contains the same PTC as that of c.362 C > A, but which is targeted by NMD. Meanwhile, the dsV2 transcripts from both c.182 A > G and c.356 C > G mutants have the same PTC, yet only c.356 C > G is subject to NMD. Given that expression of csV1 and dsV2 is reciprocal, cells with NMD-sensitive dsV2 variants, including c.318 C > G and c.356 C > G mutations, also show undetectable csV1 expression, suggesting that severe impairment of constitutive splicing correlates with increased likelihood of NMD targeting. Conversely, cells harboring the c.182 A > G and c.362 C > A mutations, both of which evade NMD, exhibit moderate to robust csV1 expression. Collectively, these data suggest that the targeting of PTCs by NMD may depend on the effect of the mutation on constitutive splicing, with mutations that severely impair constitutive splicing being likely candidates for NMD targets. These findings imply that csV1 expression levels correlate with the NMD targeting of dsV2 and may serve as a predictive indicator for NMD susceptibility.

The expression of *TP53* csV1 is crucial for p53 protein synthesis, which governs key functions, such as growth arrest and apoptosis. In cells with c.318 C > G and c.356 C > G mutations, the lack of csV1 expression leads to p53 protein deficiency and loss of p53 function, classifying these mutations as loss-of-function. Remarkably, splicing modulators can restore p53 protein expression in these loss-of-function variants, resulting in recovery of p53-dependent transcriptional activity.

In contrast, the c.182 A > G mutation results in partial loss of function, with csV1 expression reduced to 25% of the wildtype. This aligns with the haploinsufficiency model, in which one functional *TP53* allele provides limited but sufficient p53 activity [44]. AMO treatment effectively masked the novel 5’SS introduced by c.182 A > G, enhancing csV1 expression and restoring p53-dependent transcriptional responses.

Unlike other mutations for which functions are improved or restored by additional AMO treatment, the c.362 C > A mutant cells present a more complex phenotype. Although AMO treatment had minimal impact on splicing patterns or apoptosis, the mutant p53 protein retained partial function. Intriguingly, c.362 C > A mutation activates oncogenic pathways, including KRAS signaling and estrogen responses, in addition to canonical p53 target genes. This duality suggests a gain-of-function phenotype arising from amino acid changes, rather than splicing alterations, emphasizing the diverse consequences of *TP53* missense mutations.

Overall, our results indicate that splicing abnormalities caused by *TP53* mutations manifest as differences in the degree and alterations of p53 function. Mutations with mild defects, such as c.362 C > A, may permit near-normal expression of mutant p53 proteins, allowing novel gain-of-function activities to emerge. Conversely, mutations with severe splicing alteration, such as c.318 C > G and c.356 C > G, produce p53-deficient states, which are sensitive to therapeutic rescue via AMO-mediated splicing correction. Importantly, this p53 restoration re-establishes its ability to regulate critical pathways involved in cell fate determination, including apoptosis and cell cycle arrest. These findings suggest that the therapeutic efficacy of splicing-correction strategies such as AMO treatment may depend on the baseline abundance of correctly spliced transcripts. Mutations that severely disrupt authentic splicing and result in minimal baseline *TP53* expression may provide a greater therapeutic window for functional restoration than variants that retain substantial levels of correctly spliced transcripts.

In a broader clinical context, these findings may have implications for both the treatment of sporadic cancers and the management of individuals with germline *TP53* mutations, such as those with Li-Fraumeni syndrome (LFS). In sporadic cancers, where *TP53* mutations are frequently acquired, splicing-correction strategies may provide a means to restore p53 function in established tumors. In contrast, in the context of LFS, such approaches may have potential for early intervention or cancer prevention by maintaining functional p53 activity in high-risk individuals. However, given that p53 activation is tightly linked to cellular stress responses, including DNA damage, the therapeutic application of these strategies may be more immediately relevant to the treatment of active cancers, while their role in cancer prevention would require careful evaluation of long-term safety and efficacy.

## Electronic Supplementary Material

Below is the link to the electronic supplementary material.

Supplementary Material 1.

Supplementary Material 2.

Supplementary Material 3.

Supplementary Material 4.

Supplementary Material 5.

Supplementary Material 6.

Supplementary Material 7.

Supplementary Material 8.

Supplementary Material 9.

Supplementary Material 10.

Supplementary Material 11.

## Data Availability

RNA-seq data have been deposited at GEO: GSE297670, are publicly available, and can be accesses at: [https://www.ncbi.nlm.nih.gov/geo/query/acc.cgi?&acc=GSE297670](https:/www.ncbi.nlm.nih.gov/geo/query/acc.cgi?&acc=GSE297670) .
