# Supplementary material for: Splice-altering TP53 missense mutations as drivers of dysfunction and targets for RNA-based therapy
Source: Stem Cell Res Ther. 2026 Jul 13;17:358. doi: 10.1186/s13287-026-05176-1 (PMC13644261; doi:10.1186/s13287-026-05176-1)
Supplement: Supplementary file 1 [file 13287_2026_5176_MOESM10_ESM.docx]

## Supplementary Table 6. Oligonucleotide sequences for TP53 mutant minigene construction

| **minigene** | **primer name** | **sequence (5**′ **to 3**′**)** |
| --- | --- | --- |
| Universal | 5cF | (Phosphate) CACCGAATTCTGCAGAT CTGGATCCCCACTTTTCCTCTTG |
|  | 5cR | (Phosphate) GCCGCCACTGTGCTGGAT GCCTGGGGACCCTGGG |
| c.178C>A | 178R | ACCTGGGTCTTCAGTGAACC |
|  | 178F | ACTGAAGACCCAGGTACAGATGAAGCTCCCAGAATGCC |
| c.182A>G | 182R | CTGGACCTGGGTCTTCAGTG |
|  | 182F | GAAGACCCAGGTCCAGGTGAAGCTCCCAGAATGCCAGAG |
| c.318C>G | 318R | CTGCCCTGGTAGGTTTTCTGG |
|  | 318F | AAAACCTACCAGGGCAGGTACGGTTTCCGTCTGGGCTTC |
| c.356C>G | 356R | CTGTCCCAGAATGCAAGAAGC |
|  | 356F | TTGCATTCTGGGACAGGCAAGTCTGTGACTTGCACGGTC |
| c.362C>A | 362R | ACTTGGCTGTCCCAGAATGCAAG |
|  | 362F | TCTGGGACAGCCAAGTATGTGACTTGCACGGTCAGTTGC |
| c.551A>G | 551F1 | (Phosphate) CCGAATTCTGCA GATATC ATCCCCACTTTTCCTCTTGC |
|  | 551R1 | TGACACTCACAGTTTCCATAGGTCTGAAAATGTTTCC |
|  | 551F2 | TATGGAAACTGTGAGTGTCAGTTGCCCTGAGGGGC |
|  | 551R2 | CATCGCTACCTGAGCAGCGCTCATGGTG |
|  | 551F3 | CGCTGCTCAGGTAGCGATGGTGAGCAGCTG |
|  | 551R3 | (Phosphate) GCCACTGTGCTG GATATC GGTAAGAGGTGGGCC |
| c.922C>G | 922F1 | (Phosphate) CCGAATTCTGCA GATATC ATCCCCACTTTTCCTCTTGCAG |
|  | 922R1 | CAGACACTCACAGTTTCCATAGGTCTGAAAATGTTTCC |
|  | 922F2 | ATGGAAACTGTGAGTGTCTGGTTTGCAACTGGGGTC |
|  | 922R2 | TGTTGGGCACTGCTAGGAAAGAGGCAAGGAAAGG |
|  | 922F3 | CTTTCCTAGCAGTGCCCAACAACACCAGCTC |
|  | 922R3 | (Phosphate) GCCACTGTGCTG GATATCACTGCCCCCTGATGG |
| Sequencing | CMV-F | CGCAAATGGGCGGTAGGCGTG |
|  | BGH-CL-R | CGATGCAATTTCCTCATTTT |
|  | p53-intP-F | TGGCCCCTGTCATCTTCTG |
|  | 773-intP-F | GGAGAATGGCGTGAACCTG |
|  | 669-intP-F1 | ACAAAAAATTAGCCAGGCATGG |
|  | 669-intP-F2 | CAGGCCTCTGATTCCTCAC |
