# Supplementary material for: Splice-altering TP53 missense mutations as drivers of dysfunction and targets for RNA-based therapy
Source: Stem Cell Res Ther. 2026 Jul 13;17:358. doi: 10.1186/s13287-026-05176-1 (PMC13644261; doi:10.1186/s13287-026-05176-1)
Supplement: Supplementary file 2 [file 13287_2026_5176_MOESM11_ESM.docx]

## Supplementary Table 7. Oligonucleotide sequences and Antisense Morpholino Oligos (AMOs) used in this study

| **analysis** | **primer name/AMO** | **sequence (5**′ **to 3**′**)** |
| --- | --- | --- |
| **Quantitative real-time PCR (SYBR)** | GAPDHF | CCTCAACGACCACTTTGTCAAG |
|  | GAPDHR | TCTTCCTCTTGTGCTCTTGCTG |
|  | 182csV1F | TGAAGCTCCCAGAATGCCA |
|  | 182dsV2F | AAGACCCAGGTCCAGTACTCC |
|  | 318csV1F | TGTGACTTGCACGTACTCCC |
|  | 318dsV2F | AACCTACCAGGGCAGTACTCC |
|  | 356csV1F/362csV1F | GCAAGTCTGTGACTTGCACG |
|  | 356dsV2F | TTGCATTCTGGGACAGTACTC |
|  | 362dsV2F | TCTGGGACAGCCAATACTCC |
|  | 182 (V1+V2) F | GCAATGGATGATTTGATGCTGT |
|  | 182 (V1+V2) R | TTGGCAAAACATCTTGTTGAGG |
|  | 318 (V1+V2) F | CCTGTCATCTTCTGTCCCTTC |
|  | 356 (V1+V2) F/ 362 (V1+V2) F | GTCCCTTCCCAGAAAACCTAC |
|  | 318E5R^a^ | TGTGGAATCAACCCACAGC |
|  | CDKN1AF | ACCTGGAGACTCTCAGGGTCG |
|  | CDKN1AR | TTAGGGCTTCCTCTTGGAGAAGAT |
|  | BBC3F | GACGACCTCAACGCACAG |
|  | BBC3R | CTAATTGGGCTCCATCTC |
| **Minigene assay** | P1 | GCAATGGATGATTTGATGCTGT |
|  | P2 | CCTGTCATCTTCTGTCCCTTCC |
|  | P3 | GTCCCTTCCCAGAAAACCTACC |
|  | P4 | TTGGCAAAACATCTTGTTGAGG |
|  | P5 | TGTGGAATCAACCCACAGC |
|  | P6 | ACAAGCAGTCACAGCACATGAC |
|  | P7 | TTCCACTCGGATAAGATGCTGA |
|  | P8 | GGAGCACTAAGCGAGTGCC |
|  | P9 | AAGGGTGAAATATTCTCCATCCAG |
| **Genotype** | G1 | CAGAAGCTTTGCAGTGAAAGGA |
|  | G2 | CTCCTCTTCAGACCTGAAGTTCC |
|  | G3 | GTAGCTGGGATTACAGGCGAGT |
| **AMOs** | 182 AMO 1 | TCACCTGGACCTGGGTCTTCAGTGA |
|  | 182 AMO 2 | TTCACCTGGACCTGGGTCTTCAGTG |
|  | 318 AMO 1 | GGAAACCGTACCTGCCCTGGTAGGT |
|  | 318 AMO 2 | CGTACCTGCCCTGGTAGGTTTTCTG |
|  | 318 AMO 3 | GTACCTGCCCTGGTAGGTTTTCTGG |
|  | 356 AMO 1 | TTGCCTGTCCCAGAATGCAAGAAGC |
|  | 356 AMO 2 | CTTGCCTGTCCCAGAATGCAAGAAG |
|  | 362 AMO1 | ATACTTGGCTGTCCCAGAATGCAAG |
|  | 362 AMO 2 | CATACTTGGCTGTCCCAGAATGCAA |

^a^318E5R was commonly used as a reverse primer for quantitative real-time PCR.
