# Supplementary material for: Splice-altering TP53 missense mutations as drivers of dysfunction and targets for RNA-based therapy
Source: Stem Cell Res Ther. 2026 Jul 13;17:358. doi: 10.1186/s13287-026-05176-1 (PMC13644261; doi:10.1186/s13287-026-05176-1)
Supplement: Supplementary file 3 [file 13287_2026_5176_MOESM5_ESM.docx]

## Supplementary Table 1. Creation, loss, or change of splice sites predicted by NetGene2

| **Creation of 35 splice sites** | | | | **Loss of 13 splice sites** | | | **Changes in probability of 41 splice sites** | | | |
| --- | --- | --- | --- | --- | --- | --- | --- | --- | --- | --- |
| **mutation** | **5´SS^a^** | **3´SS^b^** | **Confi**  **(≥0.3)** | **Mutation** | **SD^c^** | **SA^d^** | **mutation** | **SD** | **SA** | **nor^e^ 🡪mut^f^** |
| c.40C>G | O |  | 0.79 | c.559G>A | O |  | c.557A>T | O |  | 0.94🡪0.80 |
| c.178C>A | O |  | 0.89 | c.559G>T | O |  | c.557A>G | O |  | 0.94🡪0.82 |
| c.178C>G | O |  | 0.30 | c.671A>C | O |  | c.559G>C | O |  | 0.94🡪0.55 |
| c.182A>G | O |  | 0.60 | c.671A>G | O |  | c.615T>C | O |  | 0.31🡪0.55 |
| c.318C>G | O |  | 0.47 | c.671A>T | O |  | c.616T>A | O |  | 0.31🡪0.55 |
| c.351G>T | O |  | 0.52 | c.672G>C | O |  | c.618G>C | O |  | 0.31🡪0.55 |
| c.356C>G | O |  | 0.88 | c.672G>T | O |  | c.625A>G | O |  | 0.31🡪0.55 |
| c.359A>G | O |  | 0.42 | c.782G>T | O |  | c.625A>C | O |  | 0.31🡪0.55 |
| c.362C>A | O |  | 0.90 | c.992A>T | O |  | c.626G>A | O |  | 0.31🡪0.39 |
| c.363T>A | O |  | 0.70 | c.993G>C | O |  | c.640C>G | O |  | 0.31🡪0.55 |
| c.516T>A | O |  | 0.53 | c.993G>T | O |  | c.641A>G | O |  | 0.31🡪0.55 |
| c.516T>G | O |  | 0.53 | c.562C>T |  | O | c.642T>G | O |  | 0.31🡪0.55 |
| c.551A>G | O |  | 0.94 | c.568C>T |  | O | c.643A>G | O |  | 0.31🡪0.55 |
| c.668C>G | O |  | 0.70 | - | - | - | c.644G>T | O |  | 0.31🡪0.55 |
| c.773A>T | O |  | 0.55 | - | - | - | c.670G>A | O |  | 0.31🡪0.70 |
| c.870C>T | O |  | 0.37 | - | - | - | c.780C>T | O |  | 0.90🡪0.71 |
| c.879G>T | O |  | 0.86 | - | - | - | c.781A>C | O |  | 0.90🡪0.71 |
| c.895C>G | O |  | 0.31 | - | - | - | c.781A>G | O |  | 0.90🡪0.71 |
| c.905G>T | O |  | 0.82 | - | - | - | c.781A>T | O |  | 0.90🡪0.70 |
| c.906G>T | O |  | 0.91 | - | - | - | c.782G>A | O |  | 0.90🡪0.55 |
| c.1054G>T | O |  | 0.71 | - | - | - | c.782G>C | O |  | 0.90🡪0.55 |
| c.1055A>G | O |  | 0.24 | - | - | - | c.919G>T | O |  | 1.00🡪0.70 |
| c.1064C>T | O |  | 0.70 | - | - | - | c.1032G>A | O |  | 0.86🡪0.83 |
| c.1068G>T | O |  | 1.00 | - | - | - | c.560G>A |  | O | 0.97🡪0.77 |
| c.1073A>T | O |  | 0.55 | - | - | - | c.560G>T |  | O | 0.97🡪0.19 |
| c.1082G>T | O |  | 0.95 | - | - | - | c.565G>C |  | O | 0.97🡪0.94 |
| c.1083G>T | O |  | 0.31 | - | - | - | c.565G>T |  | O | 0.97🡪0.70 |
| c.395A>G |  | O | 0.79 | - | - | - | c.571C>T |  | O | 0.56🡪0.74 |
| c.591G>A |  | O | 0.82 | - | - | - | c.577C>A |  | O | 0.56🡪0.95 |
| c.704A>G |  | O | 0.82 | - | - | - | c.577C>G |  | O | 0.56🡪0.95 |
| c.729G>A |  | O | 0.52 | - | - | - | c.578A>G |  | O | 0.56🡪0.83 |
| c.794T>A |  | O | 0.56 | - | - | - | c.578A>T |  | O | 0.56🡪0.77 |
| c.834T>A |  | O | 0.79 | - | - | - | c.579T>G |  | O | 0.56🡪0.82 |
| c.922C>G |  | O | 0.95 | - | - | - | c.580C>G |  | O | 0.56🡪0.77 |
| c.955A>C |  | O | 0.53 | - | - | - | c.581T>G |  | O | 0.56🡪0.76 |
| - | - | - | - | - | - | - | c.582T>C |  | O | 0.56🡪0.83 |
| - | - | - | - | - | - | - | c.582T>G |  | O | 0.56🡪0.83 |
| - | - | - | - | - | - | - | c.582T>A |  | O | 0.56🡪0.76 |
| - | - | - | - | - | - | - | c.586C>G |  | O | 0.56🡪0.74 |
| - | - | - | - | - | - | - | c.797G>C |  | O | 0.97🡪0.81 |
| - | - | - | - | - | - | - | c.952C>G |  | O | 0.19🡪0.83 |

^a^5´SS, 5´Splice Site; ^b^3´SS, 3´Splice Site; ^c^SD, Splicing Donor; ^d^SA, Splicing Acceptor; ^e^nor, normal; ^f^mut, mutation.
