# Supplementary material for: Splice-altering TP53 missense mutations as drivers of dysfunction and targets for RNA-based therapy
Source: Stem Cell Res Ther. 2026 Jul 13;17:358. doi: 10.1186/s13287-026-05176-1 (PMC13644261; doi:10.1186/s13287-026-05176-1)
Supplement: Supplementary file 4 [file 13287_2026_5176_MOESM6_ESM.docx]

**Supplementary Table 2. Creation or affection of splice sites predicted by Mutpred**

| ***TP53* Mutation** | | | | **Splice site** | | **Mutpred** | |
| --- | --- | --- | --- | --- | --- | --- | --- |
| **CDS^a^** | **AA^b^** | **Type** | **Distribution** | **5´** | **3´** | **Confid** |  |
| c.40C>G | L14V | mis^e^ | Exon 2 | O |  | 0.741 |  |
| c.72A>T | K24N | mis | Exon 2 (SD^c^) |  |  | 0.686 |  |
| c.76C>T | L26F | mis | Exon 3 (SA^d^) |  |  | 0.64 |  |
| c.97T>A | S33T | mis | Exon 4 |  |  | 0.759 |  |
| c.97T>C | S33P | mis | Exon 4 |  |  | 0.76 |  |
| c.98C>T | S33F | mis | Exon 4 |  |  | 0.704 |  |
| c.116C>T | A39V | mis | Exon 4 | O |  | 0.725 |  |
| c.178C>A | P60T | mis | Exon 4 | O |  | 0.902 |  |
| c.178C>G | P60A | mis | Exon 4 | O |  | 0.707 |  |
| c.182A>G | D61G | mis | Exon 4 | O |  | 0.879 |  |
| c.314G>T | G105V | mis | Exon 4 | O |  | 0.839 |  |
| c.318C>G | S106R | mis | Exon 4 | O |  | 0.97 |  |
| c.325T>A | F109I | mis | Exon 4 | O |  | 0.696 |  |
| c.351G>T | G117G | syn^f^ | Exon 4 | O |  | 0.76 |  |
| c.356C>G | A119G | mis | Exon 4 | O |  | 0.47 |  |
| c.359A>G | K120R | mis | Exon 4 | O |  | 0.848 |  |
| c.373A>C | T125P | mis | Exon 4 | O |  | 0.738 |  |
| c.373A>G | T125A | mis | Exon 4 (SD) |  |  | 0.749 |  |
| c.374C>A | T125K | mis | Exon 4 (SD) |  |  | 0.757 |  |
| c.374C>G | T125R | mis | Exon 4 (SD) |  |  | 0.801 |  |
| c.374C>T | T125M | mis | Exon 4 (SD) |  |  | 0.787 |  |
| c.376T>A | Y126N | mis | Exon 5 (SA) |  |  | 0.756 |  |
| c.376T>C | Y126H | mis | Exon 5 (SA) |  |  | 0.755 |  |
| c.376T>G | Y126D | mis | Exon 5 (SA) |  |  | 0.747 |  |
| c.377A>C | Y126S | mis | Exon 5 (SA) |  |  | 0.716 |  |
| c.377A>G | Y126C | mis | Exon 5 (SA) |  |  | 0.699 |  |
| c.377A>T | Y126F | mis | Exon 5 (SA) |  |  | 0.717 |  |
| c.395A>G | K132R | mis | Exon 5 |  | O | 0.39 |  |
| c.397A>G | M133V | mis | Exon 5 | O |  | 0.641 |  |
| c.433C>G | L145V | mis | Exon 5 | O |  | 0.907 |  |
| c.434T>G | L145R | mis | Exon 5 | O |  | 0.843 |  |
| c.516T>A | V172V | syn | Exon 5 | O |  | 0.79 |  |
| c.516T>G | V172V | syn | Exon 5 | O |  | 0.32 |  |
| c.522G>T | R174S | mis | Exon 5 | O |  | 0.816 |  |
| c.551A>G | D184G | mis | Exon 5 | O |  | 0.953 |  |
| c.557A>G | D186G | mis | Exon 5(SD) |  |  | 0.76 |  |
| c.557A>T | D186V | mis | Exon 5 (SD) |  |  | 0.766 |  |
| c.558T>A | D186E | mis | Exon 5 (SD) |  |  | 0.763 |  |
| c.559G>A | G187S | mis | Exon 5 (SD) |  |  | 0.826 |  |
| c.559G>C | G187R | mis | Exon 5 (SD) |  |  | 0.793 |  |
| c.559G>T | G187C | mis | Exon 5 (SD) |  |  | 0.807 |  |
| c.560G>A | G187D | mis | Exon 6 (SA) |  |  | 0.811 |  |
| c.560G>C | G187A | mis | Exon 6 (SA) |  |  | 0.808 |  |
| c.560G>T | G187V | mis | Exon 6 (SA) |  |  | 0.831 |  |
| c.577C>T | H193Y | mis | Exon 6 |  |  | 0.633 |  |
| c.591G>A | V197V | syn | Exon 6 |  | O | 0.59 |  |
| c.611A>G | E204G | mis | Exon 6 |  |  | 0.824 |  |
| c.619G>T | D207Y | mis | Exon 6 |  |  | 0.638 |  |
| c.620A>G | D207G | mis | Exon 6 |  |  | 0.649 |  |
| c.627A>T | R209S | mis | Exon 6 |  |  | 0.665 |  |
| c.646G>A | V216M | mis | Exon 6 |  |  | 0.627 |  |
| c.650T>A | V217E | mis | Exon 6 |  |  | 0.625 |  |
| c.656C>G | P219R | mis | Exon 6 |  |  | 0.643 |  |
| c.668C>G | P223R | mis | Exon 6 | O |  | 0.652 |  |
| c.670G>A | E224K | mis | Exon 6 (SD) |  |  | 0.843 |  |
| c.670G>C | E224Q | mis | Exon 6 (SD) |  |  | 0.773 |  |
| c.671A>C | E224A | mis | Exon 6 (SD) |  |  | 0.83 |  |
| c.671A>G | E224G | mis | Exon 6 (SD) |  |  | 0.868 |  |
| c.671A>T | E224V | mis | Exon 6 (SD) |  |  | 0.804 |  |
| c.672G>C | E224D | mis | Exon 6 (SD) |  |  | 0.827 |  |
| c.672G>T | E224D | mis | Exon 6 (SD) |  |  | 0.833 |  |
| c.673G>A | V225I | mis | Exon 7 (SA) |  |  | 0.833 |  |
| c.673G>C | V225L | mis | Exon 7 (SA) |  |  | 0.828 |  |
| c.673G>T | V225F | mis | Exon 7 (SA) |  |  | 0.837 |  |
| c.674T>A | V225D | mis | Exon 7 (SA) |  |  | 0.757 |  |
| c.674T>C | V225A | mis | Exon 7 (SA) |  |  | 0.801 |  |
| c.674T>G | V225G | mis | Exon 7 (SA) |  |  | 0.8 |  |
| c.685T>G | C229G | mis | Exon 7 | O |  | 0.844 |  |
| c.704A>G | N235S | mis | Exon 7 |  | O | 0.35 |  |
| c.712T>G | C238G | mis | Exon 7 |  |  | 0.811 |  |
| c.729G>A | M243I | mis | Exon 7 |  | O | 0.46 |  |
| c.739A>T | N247Y | mis | Exon 7 |  |  | 0.733 |  |
| c.773A>T | E258V | mis | Exon 7 | O |  | 0.863 |  |
| c.781A>G | S261G | mis | Exon 7 (SD) |  |  | 0.823 |  |
| c.781A>T | S261C | mis | Exon 7 (SD) |  |  | 0.806 |  |
| c.782G>A | S261N | mis | Exon 7 (SD) |  |  | 0.822 |  |
| c.782G>C | S261T | mis | Exon 7 (SD) |  |  | 0.823 |  |
| c.782G>T | S261I | mis | Exon 7 (SD) |  |  | 0.832 |  |
| c.783T>A | S261R | mis | Exon 8 (SA) |  |  | 0.954 |  |
| c.783T>G | S261R | mis | Exon 8 (SA) |  |  | 0.90 |  |
| c.784G>A | G262S | mis | Exon 8 (SA) |  |  | 0.86 |  |
| c.784G>C | G262R | mis | Exon 8 (SA) |  |  | 0.811 |  |
| c.784G>T | G262C | mis | Exon 8 (SA) |  |  | 0.865 |  |
| c.788A>G | N263S | mis | Exon 8 |  | O | 0.617 |  |
| c.789T>G | N263K | mis | Exon 8 |  | O | 0.815 |  |
| c.794T>A | L265Q | mis | Exon 8 |  | O | 0.40 |  |
| c.807C>G | S269R | mis | Exon 8 | O |  | 0.902 |  |
| c.834T>A | P278P | syn | Exon 8 |  | O | 0.35 |  |
| c.870C>T | R290R | mis | Exon 8 | O |  | 0.87 |  |
| c.879G>T | G293G | syn | Exon 8 | O |  | 0.91 |  |
| c.895C>G | L299V | mis | Exon 8 | O |  | 0.728 |  |
| c.905G>T | G302G | syn | Exon 8 | O |  | 0.58 |  |
| c.906G>T | G302V | mis | Exon 8 | O |  | 0.96 |  |
| c.917G>A | R306Q | mis | Exon 8 (SD) |  |  | 0.793 |  |
| c.917G>C | R306P | mis | Exon 8 (SD) |  |  | 0.78 |  |
| c.917G>T | R306L | mis | Exon 8 (SD) |  |  | 0.864 |  |
| c.919G>A | A307T | mis | Exon 8 (SD) |  |  | 0.837 |  |
| c.919G>C | A307P | mis | Exon 8 (SD) |  |  | 0.843 |  |
| c.919G>T | A307S | mis | Exon 8 (SD) |  |  | 0.853 |  |
| c.920C>T | A307V | mis | Exon 9 (SA) |  |  | 0.823 |  |
| c.922C>A | L308M | mis | Exon 9 |  | O | 0.7 |  |
| c.922C>G | L308V | mis | Exon 9 |  | O | 0.751 |  |
| c.955A>C | K319Q | mis | Exon 9 |  |  | 0.25 |  |
| c.957G>T | K319N | mis | Exon 9 |  |  | 0.63 |  |
| c.970G>T | D324Y | mis | Exon 9 | O |  | 0.702 |  |
| c.974G>T | G325V | mis | Exon 9 | O |  | 0.655 |  |
| c.991C>G | Q331E | mis | Exon 9 (SD) |  |  | 0.803 |  |
| c.992A>C | Q331P | mis | Exon 9 (SD) |  |  | 0.861 |  |
| c.992A>G | Q331R | mis | Exon 9 (SD) |  |  | 0.869 |  |
| c.992A>T | Q331L | mis | Exon 9 (SD) |  |  | 0.863 |  |
| c.993G>C | Q331H | mis | Exon 9 (SD) |  |  | 0.826 |  |
| c.993G>T | Q331H | mis | Exon 9 (SD) |  |  | 0.862 |  |
| c.994A>G | I332V | mis | Exon 10 (SA) |  |  | 0.911 |  |
| c.994A>T | I332F | mis | Exon 10 (SA) |  |  | 0.832 |  |
| c.995T>A | I332N | mis | Exon 10 (SA) |  | O | 0.763 |  |
| c.995T>C | I332T | mis | Exon 10 (SA) |  | O | 0.756 |  |
| c.995T>G | I332S | mis | Exon 10 (SA) |  | O | 0.785 |  |
| c.1003C>G | R335G | mis | Exon 10 | O |  | 0.867 |  |
| c.1006G>A | E336K | mis | Exon 10 | O |  | 0.749 |  |
| c.1009C>T | R337C | mis | Exon 10 | O |  | 0.816 |  |
| c.1018A>G | M340V | mis | Exon 10 | O |  | 0.626 |  |
| c.1054G>T | D352Y | mis | Exon 10 | O |  | 0.742 |  |
| c.1064C>T | A355V | mis | Exon 10 | O |  | 0.799 |  |
| c.1068G>T | G356G | syn | Exon 10 | O |  | 0.97 |  |
| c.1073A>T | E358V | mis | Exon 10 | O |  | 0.637 |  |
| c.1082G>T | G361V | mis | Exon 10 | O |  | 0.785 |  |
| c.1083G>T | G361G | mis | Exon 10 | O |  | 0.68 |  |
| c.1100G>A | S367N | mis | Exon 10 (SD) |  |  | 0.813 |  |
| c.1102C>G | H368D | mis | Exon 11 (SA) |  |  | 0.636 |  |
| c.1123C>A | Q375K | mis | Exon 11 | O |  | 0.857 |  |

^a^CDS, coding DNA sequence; ^b^AA, amino acid; ^c^SD, splicing donor; ^d^SA, splicing acceptor; ^e^mis, missense; ^f^syn, synonymous.
